# Pharmacogenetics of antidepressant response: a focused review on CYP2C19, CYP2D6, SLC6A4, and HTR2A polymorphisms

**DOI:** 10.3389/fphar.2026.1773677

**Published:** 2026-04-30

**Authors:** Luiza Blambila, Poliana Sabei, Vitória Gomes Raulino, Marcos Edgar Herkenhoff

**Affiliations:** Laboratory of Molecular Genetics, Center for Higher Education of the Southern Region (CERES), Santa Catarina State University (UDESC), Laguna, Santa Catarina, Brazil

**Keywords:** antidepressants, drug response, genetic polymorphism, personalized medicine, pharmacogenetics, serotonergic system

## Abstract

Pharmacogenetics has redefined the understanding of antidepressant response by demonstrating that genetic variability profoundly influences both drug efficacy and safety. This review synthesizes current evidence on the impact of pharmacokinetic and pharmacodynamic polymorphisms in guiding antidepressant therapy, focusing on the cytochrome P450 enzymes CYP2C19 and CYP2D6, as well as the serotonergic genes SLC6A4 and HTR2A. A comprehensive literature search in NCBI and Google Scholar (2017–2024) identified recent meta-analyses and clinical studies evaluating genotype–phenotype associations in patients treated with selective serotonin reuptake inhibitors (SSRIs). Findings indicate that *CYP2C19* and *CYP2D6* polymorphisms markedly affect plasma concentrations, therapeutic outcomes, and adverse-event risk—where poor metabolizers exhibit increased efficacy but greater toxicity, while ultrarapid metabolizers show reduced therapeutic response. Likewise, functional variants such as *SLC6A4* (5-HTTLPR) and *HTR2A* modulate serotonin transporter availability and receptor sensitivity, influencing clinical improvement and tolerability, especially in interaction with environmental stressors. The integration of these genetic markers into conceptual clinical frameworks enables more rational antidepressant selection, personalized dosing, and minimization of adverse reactions, demonstrating that both pharmacokinetic and pharmacodynamic polymorphisms jointly contribute to antidepressant efficacy within a stepwise precision-medicine approach.

## 1 Introduction

Depression is currently considered one of the most disabling psychiatric conditions, affecting millions of individuals and representing a significant burden for public health systems (Simon et al., 2024). In Brazil, the prevalence of antidepressant use reflects the high incidence of depressive disorders in the population, with consumption estimates exceeding 4%, particularly among women and the elderly (Tiguman et al., 2023). The treatment of depression commonly involves the use of antidepressants, most notably selective serotonin reuptake inhibitors (SSRIs), due to their favorable efficacy and tolerability profiles (Ghaffari Darab et al., 2020; Marasine et al., 2021). Although adverse effects are associated with this type of treatment, the therapeutic benefits are evident (Köhler-Forsberg et al., 2023). In this context, the use of antidepressants is essential in the management of mood disorders.

This review aims to understand how genetic polymorphisms influence the action of antidepressants, as well as to identify relevant genetic variants and how they affect treatment response. The present study is a narrative literature review, aiming to compile and analyze available data on the influence of genetic polymorphisms in serotonergic genes on antidepressant response. The search for scientific articles was conducted using databases such as NCBI and Google Scholar, employing descriptors related to the topic, including “Antidepressants,” “Depression,” “Pharmacogenomics,” “Pharmacogenetics,” “Polymorphism,” “Serotonergic,” in addition to specific target genes such as SLC6A4, HTR2A, CYP2D6, and CYP2C19, in order to encompass the most relevant studies.

Articles published in the past 30 years that directly addressed the relationship between genetic polymorphisms and the efficacy, metabolism, or clinical response to antidepressants were considered, with emphasis on selective serotonin reuptake inhibitors (SSRIs). The selection of studies was guided by their relevance to the central theme, methodological quality, and the full-text availability of the content. After screening and reading the selected articles, the data were organized and synthesized based on recurring thematic categories, considering aspects such as the gene studied, the type of polymorphism described, the antidepressant class involved, the population evaluated, and the clinical implications observed.

The adopted methodology aims to ensure coherence, consistency, and relevance in the analysis, providing a comprehensive understanding of the impact of genetic polymorphisms on antidepressant response, and highlighting the growing importance of pharmacogenetics in current clinical practice.

This narrative review was conducted through a structured search of the PubMed/MEDLINE and Google Scholar databases. We used combinations of the terms “depression”, “antidepressants”, “pharmacogenetics”, “pharmacogenomics”, “CYP2D6”, “CYP2C19”, “SLC6A4”, and “HTR2A″ to identify relevant human studies published between January 1994 and March 2026. We included original clinical research, meta-analyses, and major guidelines that examined the association between genetic variants in these genes and antidepressant pharmacokinetics, efficacy, or tolerability in adults or pediatric populations. We excluded case reports, animal studies, non-systematic opinion pieces, and papers not available in English, Portuguese, or Spanish. The initial search retrieved approximately 39,116 records; after screening titles and abstracts for relevance, and reviewing full texts where necessary, we retained 200 key studies and guideline documents that directly addressed the relationship between these pharmacogenetic markers and antidepressant treatment outcomes.

## 2 General mechanism of action of antidepressants

Currently, antidepressants are primarily classified based on their pharmacological mechanisms, which aim to enhance monoaminergic neurotransmission, especially within the noradrenergic and serotonergic systems (Berton and Nestler, 2021). This modulation occurs through different strategies, such as inhibiting neurotransmitter metabolism, blocking reuptake at presynaptic terminals, and regulating autoreceptors that control neurotransmitter release and synaptic sensitivity. These actions lead to increased concentration and prolonged activity of neurotransmitters that are critical for mood regulation (Berton and Nestler, 2021; Cui et al., 2024).

Monoamine oxidase inhibitors (MAOIs) exert their antidepressant effects by inhibiting the enzyme monoamine oxidase (MAO), responsible for the degradation of monoaminergic neurotransmitters—such as serotonin, norepinephrine, and dopamine—within presynaptic neural and mitochondrial compartments (Tan et al., 2022). MAO has two major isoforms, MAO-A and MAO-B, which differ in their substrate specificities. The inhibition of MAO-A is particularly relevant for increasing serotonin and norepinephrine levels, neurotransmitters closely linked to the alleviation of depressive symptoms (Naoi et al., 2025; Tan et al., 2022). Moreover, reversible and selective MAOIs, such as moclobemide, have been developed to reduce the severe side effects associated with classical MAOIs—such as hypertensive crises triggered by dietary intake—thereby increasing the safety and acceptability of treatment (Tan et al., 2022; Yamada and Yasuhara, 2004).

Tricyclic antidepressants (TCAs) exert their primary therapeutic effect by blocking the presynaptic reuptake of norepinephrine and serotonin, thereby increasing synaptic concentrations and enhancing neurotransmission in these systems (Gillman, 2007). Beyond reuptake inhibition, TCAs have affinity for various postsynaptic receptors, including muscarinic cholinergic, histaminergic H1, and α1-adrenergic receptors, which contribute to their characteristic side effect profile, such as dry mouth, sedation, and orthostatic hypotension (Gillman, 2007). At the molecular level, chronic administration of TCAs leads to the desensitization of β1-adrenergic and 5-HT2 serotonergic receptors—an adaptive phenomenon thought to underlie the development of sustained antidepressant effects over time (Berton and Nestler, 2021). These adaptive changes involve intracellular signaling cascades and gene expression regulation, reflecting the complexity of these drugs’ mechanisms of action, which extend beyond the immediate increase in synaptic neurotransmitter levels (Berton and Nestler, 2021).

Selective serotonin reuptake inhibitors (SSRIs) are currently the most prescribed antidepressants due to their favorable efficacy and safety profiles. They selectively block the serotonin transporter (SERT), thereby inhibiting the reuptake of serotonin at the synaptic cleft and increasing serotonergic neurotransmission (Cipriani et al., 2018). This sustained elevation in serotonin levels induces neurochemical and neurophysiological adaptations—such as desensitization of 5-HT1A autoreceptors—which enhance the clinical antidepressant effect after several weeks of treatment (Cipriani et al., 2018; Gillman, 2007).

In addition to reuptake inhibition, SSRIs differ in their pharmacokinetic properties, including half-life, hepatic metabolism, and interactions with cytochrome P450 enzymes. These factors influence onset of action, duration of effect, and side effect profiles (Cipriani et al., 2018). For example, fluoxetine has a long half-life and active metabolites that prolong its action, whereas sertraline has moderate additional affinity for dopaminergic receptors, potentially affecting specific clinical responses (Cipriani et al., 2018).

Large-scale clinical studies, including network meta-analyses, have demonstrated that SSRIs are more effective than placebo and present an acceptable tolerability profile. However, individual differences in adverse effects and therapeutic response must be considered to guide personalized treatment choices (Cipriani et al., 2018).

## 3 Variability in antidepressant response

The therapeutic response to antidepressants is highly variable among patients, representing a significant challenge in clinical practice. Several individual factors, such as genetic and neurobiological differences, appear to influence the variability in treatment responses. According to Cipriani et al. (2018), only a portion of patients experience successful therapeutic outcomes, while others develop significant adverse effects—a phenomenon that may be associated with distinct neural substrates and genetic mutations influencing individual responses.

This heterogeneity is observed even within the same pharmacological class. Selective serotonin reuptake inhibitors (SSRIs), for example, are generally better tolerated than tricyclic antidepressants (TCAs), yet they are not free from adverse effects. SSRIs “lead to fewer adverse effects. However, they are not devoid of them, with commonly reported symptoms including headache, sweating, anxiety, agitation, gastrointestinal disturbances, fatigue, and malaise. Sexual dysfunctions, such as decreased libido, anorgasmia, and anejaculation, are also frequent” (Edinoff et al., 2021).

On the other hand, tricyclic antidepressants, such as amitriptyline and nortriptyline, while effective, are associated with a broader range of adverse effects due to their action on multiple receptors. Tricyclic antidepressants (TCAs) act on multiple neurotransmitter receptor systems—including noradrenergic, serotonergic, histaminergic, alpha-adrenergic, muscarinic, and dopaminergic pathways—which explains their broad range of side effects. These may include orthostatic hypotension, dry mouth, tremors, constipation, tachycardia, prolongation of PR and QRS intervals on the ECG, and a reduction in systolic blood pressure upon standing (Carvalho Júnior and Trevisan, 2021).

In addition to adverse effects, another factor that compromises treatment adherence is the latency period before the onset of therapeutic action. Many antidepressants, particularly SSRIs and TCAs, require a minimum period of several weeks to achieve the desired effect. According to Carvalho Júnior and Trevisan (2021), the therapeutic effects of antidepressants typically become evident only after three to four weeks of treatment, which requires gradual dose titration to minimize the occurrence of adverse effects.

Finally, therapeutic inefficacy may also be related to factors such as clinical comorbidities or intrinsic characteristics of the pathophysiology of depression, which remains incompletely understood. In this context, Richelson (2013) point out that the limited therapeutic effectiveness of antidepressant treatments may stem from several factors, such as the delay between drug administration and clinical improvement, which occurs even when effective plasma concentrations are reached. They also note that the pharmacological action sites of antidepressants may not fully correspond to the neurobiological regions implicated in depressive pathology. Furthermore, the presence of comorbid clinical conditions and incomplete understanding of the developmental mechanisms underlying depression further contribute to the reduced efficacy of these treatments.

## 4 Genetic influence on pharmacodynamics and pharmacokinetics

The therapeutic Pharmacogenetics investigates how genetic variability influences individual responses to conventional pharmacological treatments, given that genetics can interfere with both the efficacy and toxicity of a drug. Due to the considerable diversity in drug responses, that is, the fact that the same pharmacological treatment can produce varying levels of efficacy and toxicity across individuals, the application of pharmacogenetics becomes essential. This approach enables the identification of factors underlying such variability in treatment response and allows for the implementation of personalized therapy based on genetic individuality, thereby resulting in more effective, safer, and less toxic treatments (Sadee et al., 2023).

Etymologically, the term *polymorphism* derives from the Latin *polymorphism*, meaning “many” (*poly*) “forms” (*morph*). This meaning contributes to the understanding that there are multiple forms of a genomic region or allele. A defining characteristic of this term is the presence of sequence variation, which manifests in different forms among individuals (Panneerchelvam and Norazmi, 2023). Polymorphisms could differentiate individuals through genetic markers, currently known as minisatellites and microsatellites. These marker-related variations may be inherited through the process of linkage. However, these markers are not transmitted in their entirety; only some are selected during crossing over, with contributions from both parental origins—maternal and paternal—thus allowing for genetic relationships to be inferred among individuals (Bagshaw, 2017).

It is well established that patients treated with the same pharmacological therapy may exhibit different responses, including variations in drug toxicity. Identical doses may differ in terms of efficacy and adverse effects. A range of factors influence drug response, including age, ethnicity, sex, diet, health status, environment, as well as the drug’s pharmacokinetics and pharmacodynamics. Nevertheless, genetic polymorphisms stand out as a key determinant of drug response, particularly in genes encoding metabolizing enzymes, transporters, or receptors—making genetic factors crucial for both efficacy and toxicity (Sadee et al., 2023).

## 5 Metabolism of escitalopram by cytochrome P450 enzymes: pathways and clinical implications

**FIGURE 1:**
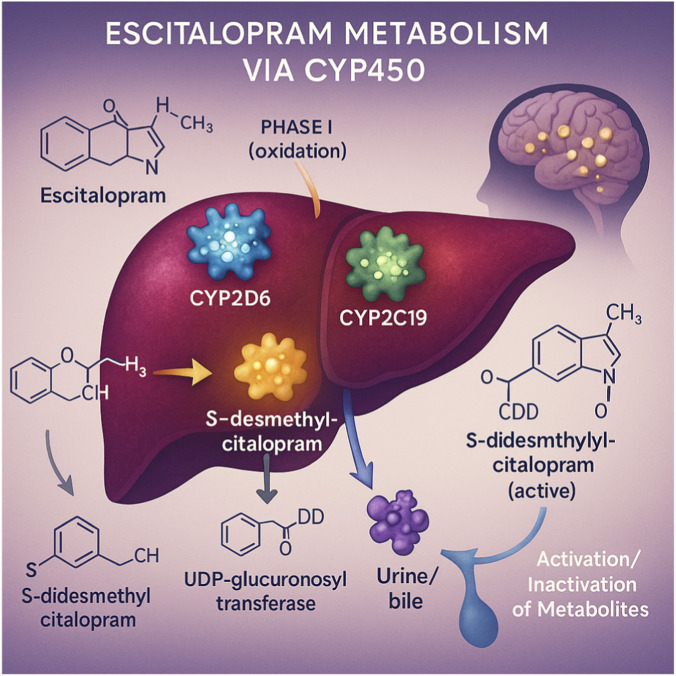
Illustration of the hepatic metabolism of escitalopram mediated by cytochrome P450 enzymes. The parent compound undergoes oxidation (Phase I) by the CYP2C19 and CYP3A4 isoenzymes, generating the active metabolite S-desmethylcitalopram, which is subsequently converted by CYP2D6 into the inactive metabolite S-didesmethylcitalopram. Conjugation (Phase II), catalyzed by UDP-glucuronosyltransferase, facilitates urinary or biliary excretion. The therapeutic effect is achieved through the inhibition of serotonin reuptake in the central nervous system.

The therapeutic Pharmacogenetics investigates how genetic variability influences individual responses Cytochrome P450 (CYP) enzymes in the liver catalyze phase I reactions by introducing functional groups into lipophilic compounds. These reactive metabolites can subsequently undergo conjugation by phase II enzymes, such as UDP-glucuronosyltransferases, forming hydrophilic conjugates that are more readily excreted in the urine or bile (Ul Amin Mohsin et al., 2024). Escitalopram, a selective serotonin reuptake inhibitor (SSRI), is widely used in the treatment of Major Depressive Disorder (MDD) and anxiety disorders (Baldwin et al., 2007) (Figure 1). It is primarily metabolized by CYP2D6, CYP2C19, and CYP3A4 (Rao, 2007). Escitalopram is first metabolized via N-desmethylation to S-desmethylcitalopram by CYP3A4 and CYP2C19 (Kobayashi et al., 1997), and this metabolite is subsequently demethylated mainly by CYP2D6 to form S-didesmethylcitalopram (von Moltke et al., 2001). Among these, escitalopram and S-desmethylcitalopram are the active metabolites with antidepressant effects, whereas S-didesmethylcitalopram is pharmacologically inactive (Kobayashi et al., 1997). Depending on CYP enzyme activity, patients taking escitalopram may exhibit varying plasma concentrations of escitalopram and its metabolites, S-desmethylcitalopram and S-didesmethylcitalopram (Reis et al., 2003).

## 6 CYP2D6 polymorphism and its clinical implications in antidepressant treatment

CYP2D6 is a particularly important and well-studied member of the CYP superfamily. In the late 1970s, the metabolism of debrisoquine, and separately sparteine, were both shown to be highly variable yet controlled by a single autosomal gene, which we now know to by CYP2D6 (Taylor et al., 2020). CYP2D6 polymorphism is one of the most clinically relevant genetic variations due to its influence on the enzymatic activity of CYP2D6, which plays a central role in the metabolism of several antidepressants (Bertilsson, 2007; Li et al., 2024; Kee et al., 2023). In this context, recent large-scale pharmacogenetic meta-analyses have provided extensive evidence on how genetic polymorphisms, particularly those in the cytochrome P450 enzyme family, modulate individual variability in antidepressant pharmacokinetics and treatment outcomes. Li et al. (2024) conducted one of the most comprehensive genomic meta-analyses to date, integrating individual-level data from thirteen clinical studies comprising 5,843 patients diagnosed with Major Depressive Disorder (MDD) of both European and East Asian ancestry. There are clinical pharmacogenomic guidelines for at least 48 CYP2D6 substrate medications, which provide therapeutic recommendations based on the predicted metabolizer phenotype (Taylor et al., 2020).

Their investigation focused on the metabolic activity of CYP2C19 and CYP2D6—two pivotal enzymes in the hepatic metabolism of antidepressants—and how genetic diversity within these loci influences treatment response. The authors utilized genome-wide genotyping and subsequent genotype imputation to infer metabolic phenotypes, classifying individuals into poor (PM), intermediate (IM), normal (NM), and rapid/ultrarapid metabolizers (RM/UM) according to CPIC guidelines (Bousman et al., 2023; Hicks et al., 2017).

This approach allowed the translation of genetic polymorphisms into predicted enzymatic activities, although with notable limitations for CYP2D6, whose structural variants—such as duplications and deletions that critically affect enzyme function—cannot be accurately imputed from standard SNP array data (Nofziger et al., 2020; Li et al., 2024).

Conversely, no robust association could be established for CYP2D6, as the imputation process failed to capture structural variants that define ultrarapid or poor metabolizers, resulting in partial phenotype misclassification and incomplete assessment of enzymatic activity (Li et al., 2024; Pratt et al., 2021). This methodological limitation underscores the necessity of using targeted sequencing approaches or pharmacogenetic-specific panels to more accurately assess CYP2D6 variation, which accounts for approximately 25% of the metabolism of clinically used drugs, including numerous antidepressants and antipsychotics (Fabbri et al., 2018; Zhou et al., 2017; Singh et al., 2025).

Recent work by Liao et al. (2024) provides strong prospective evidence for CYP2D6-guided dosing of paroxetine across East Asian and non-East Asian populations. In a cohort of 921 patients, paroxetine steady-state concentrations were approximately 2.5-fold higher in CYP2D6 poor metabolizers and 0.39-fold in ultrarapid metabolizers compared with extensive metabolizers, directly quantifying the impact of metabolic phenotype on drug exposure. These findings empirically support phenotype-aware dose adjustments to reduce adverse effects in PMs and avoid underdosing in UMs.

In pediatric populations, CYP2D6 is particularly relevant, with its activity stable from infancy through adolescence, and its polymorphisms influencing drug tolerability (e.g., risperidone, paliperidone) and symptom improvement (e.g., fluoxetine, amphetamines). A significant challenge is phenoconversion, where external factors alter the metabolic phenotype, frequently involving CYP2D6 and affecting nearly half of young individuals on psychotropic medication, potentially leading to suboptimal treatment outcomes or increased adverse effects. While the impact of CYP2D6 on antidepressant efficacy may be modest, its influence on tolerability is significant, underscoring the value of CYP2D6 genotyping to enhance treatment safety and effectiveness (Singh et al., 2025).

Asp et al. (2025) examined 407 patients treated for depression in secondary psychiatric care and found no significant overrepresentation of the CYP2D6 ultrarapid metabolizer (UM) phenotype among those with a history of suicide attempts (1.6% in attempters vs. 3.2% in non-attempters; p = 0.27), contrary to prior hypotheses linking UM status to increased suicidality due to potential treatment failure from rapid drug metabolism. Instead, factors such as bipolar disorder, personality disorders, substance use disorders, and lifetime psychotic symptoms were more prevalent in suicide attempters, suggesting that CYP2D6 genotyping may optimize pharmacological treatment for depression but holds limited relevance for assessing suicide risk in treatment-resistant cases (Asp et al., 2025).

Phenoconversion—affecting up to 46% of youth on psychotropics—as a key challenge for CYP2D6, often leading to altered metabolizer status and increased adverse effects or suboptimal outcomes, while emphasizing that CYP2D6 substrates account for a substantial portion (87.6%) of actionable pediatric prescriptions; it advocates for integrating CYP2D6 testing into guidelines to enhance safety and precision in child psychiatry, particularly amid barriers like developmental enzyme ontogeny and limited familiarity among clinicians (Singh et al., 2025).

The CYP2D6 is a crucial gene, where extensive metabolizers (EMs) possess *1 alleles associated with normal enzymatic function, while alleles such as *3, *4, *5, and *6 encode non-functional proteins. Poor metabolizers (PMs) of CYP2D6 present an increased risk of side effects, including suicidal ideation or antidepressant-induced mania during treatment. In contrast, CYP2D6 ultrarapid metabolizers (UMs) demonstrated a better response to venlafaxine treatment and higher remission rates in patients with major depressive disorders.

The CYP2D6 gene is notably polymorphic, with 133 ‘star’ alleles listed, which include SNPs, deletions, multiplications, and hybridizations with pseudogenes, and whose frequencies vary significantly between different populations (Prost and Płaziński, 2025). Structural variations such as the complete deletion of the gene (*CYP2D6*5) result in total loss of function, while duplications can increase activity (Prost and Płaziński, 2025). Clinically, these variations are crucial, as they influence the efficacy and safety of medications, with pharmacogenomic guidelines recommending dose adjustments or the choice of alternative drugs for poor, intermediate, or ultrarapid metabolizers, including in pediatric populations.

In patients with treatment resistance (TR) to antipsychotics and/or antidepressants, an increased allelic frequency of CYP2D6 *3 was observed (4.5% vs. 1.0% in controls, OR 4.5, p = 0.003). Additionally, all TR patients who possessed the slow metabolism *5 allele were women (3.8% vs. 0% in men, OR 11.6, p = 0.029) (Zhiganova and Radkova, 2025). Zhiganova and Radkova (2025) study also revealed a trend toward a decrease in the frequency of the non-functional *6 allele (0.4% vs. 1.2%) and an increase in the frequency of the CYP2D6 *1NUM gain-of-function allele (1.7% vs. 3.0%) in TR patients. These findings suggest that the presence of the non-functional CYP2D6 *3 allele may require reduced doses of CYP2D6 substrates (such as risperidone, fluvoxamine, imipramine, paroxetine) to prevent side effects. On the other hand, the use of venlafaxine in patients with reduced CYP2D6 activity may be less effective due to the decreased formation of the active metabolite desvenlafaxine.

CYP2D6 is highlighted as the main enzyme responsible for the metabolism of aripiprazole. This means that the way this enzyme functions in each person has a direct impact on the amount of aripiprazole that remains in the blood and for how long (Gettu and Saadabadi, 2022). If a patient is using aripiprazole as an adjunct for depression and is a poor metabolizer of CYP2D6, they will have higher levels of the medication, which can lead to side effects and, consequently, discontinuation (Jallaq et al., 2021).

The study by Gras et al. (2025) emphasizes the applicability of pre-emptive CYP2D6 genotyping (testing the patient’s genetics before starting treatment) to predict these risks. By identifying extreme metabolizers, physicians can adjust the aripiprazole dose from the outset, which is expected to reduce adverse events and inefficacy that would lead to treatment discontinuation, regardless of the specific psychiatric condition for which aripiprazole was prescribed.

The genetics of CYP2D6 is a crucial factor in how aripiprazole works in the body and in the likelihood of a patient continuing treatment. This is valid for all indications of aripiprazole, including its use as an antidepressant, and genotyping can be a valuable tool for personalizing treatment and improving outcomes for patients (Gras et al., 2025).

Despite the growing recognition of the clinical and economic benefits of optimizing medication use through pharmacogenomics, CYP2D6 genotyping still faces challenges in widespread implementation in clinical practice, particularly due to the complexity of identifying structural variants. However, the ongoing development of new guidelines and machine learning approaches promises to improve the interpretation of CYP2D6 genotype-phenotype relationships, aiming to translate this knowledge into tangible benefits for patients (Taylor et al., 2020).

## 7 CYP2C19 polymorphism: impact on antidepressant efficacy and remission rates

CYP2C19 is a fundamental enzyme in the metabolism of antidepressants, whose genetic variability is associated with treatment resistance, and advocates for the implementation of pharmacogenetic testing to personalize therapy (Zhiganova and Radkova, 2025). CYP2C19 is crucial in the metabolism of commonly used antidepressants in clinical practice, such as sertraline and escitalopram. Although CYP2B6 also contributes to the metabolism of sertraline, the impact of CYP2C19 is significantly more pronounced (Yan et al., 2025).

Li et al. (2024) revealed that CYP2C19 poor metabolizers exhibited a higher remission rate compared to normal metabolizers (OR = 1.46; 95% CI = 1.03–2.06; p = 0.033), suggesting that reduced metabolic capacity might lead to higher systemic exposure to active drug forms, thereby enhancing antidepressant efficacy. When analyses were restricted to antidepressants primarily metabolized by CYP2C19 (such as citalopram, escitalopram, and sertraline), the same tendency persisted, supporting a pharmacokinetic contribution of CYP2C19 to antidepressant response, though statistical significance again was not achieved (Li et al., 2024). CYP2C19 plays an important role in the efficacy and safe use of many medications. It was demonstrated how mutations in CYP2C19 influence the daily treatment of patients, leading to adverse drug reactions or therapeutic failure in many common diseases (Sienkiewicz-Oleszkiewicz and Wiela-Hojeńska, 2018).

The homozygous wild-type allele CYP2C19*1 possesses full drug metabolism capacity. Poor metabolizers generally possess two null alleles, such as *2A, *2B, *3A, *3B, *4A, *4B, *5A, *B, *6, *7, *8. Among them, CYP2C19*2 and CYP2C19*3 are the most common and the main alleles causing slow drug metabolism (Sienkiewicz-Oleszkiewicz and Wiela-Hojeńska, 2018). The *1 allele is the one with normal function, while the *2, *3, *4, *6, and *35 alleles are loss-of-function alleles (CYP2C19Null). The *17 allele is the only known gain-of-function allele, resulting in increased metabolism. Based on these genotypes, the population can be divided into metabolizer subgroups: poor (PMs), intermediate (IMs), normal (NMs), rapid (RMs), and ultrarapid (UMs) (Yan et al., 2025).

On the other hand, CYP2C19*17 showed increased gene expression and enzymatic activity, with the consequence of lack of response to certain medications (Sienkiewicz-Oleszkiewicz and Wiela-Hojeńska, 2018).

Interestingly, although allele frequencies differed substantially between populations—CYP2C19 *2 and *3 alleles being more frequent in East Asians, and *17 more common among Europeans—Li et al. (2024) found that the direction and magnitude of metabolizer effects did not vary across ancestries, suggesting that the biochemical influence of functional polymorphisms transcends population boundaries.

In the study by Yan et al. (2025), the frequencies of CYP2C19 alleles in the cohort of Han Chinese patients treated with sertraline and escitalopram were consistent with those observed in East Asian populations. For sertraline, the frequency of the *1 allele was 64.8%, *17 was 1.7%, and loss-of-function alleles were 33.5%. For escitalopram, the frequencies were *1 (63.6%), *17 (1.2%), and loss-of-function alleles (35.2%). The distribution of phenotypes showed that normal metabolizers represented 41.2%, intermediate metabolizers 45.8%, and poor metabolizers 10.6% of the total sample.

From a pharmacokinetic perspective, poor metabolizers exhibit longer drug half-life and higher plasma concentrations, potentially enhancing antidepressant efficacy but concurrently increasing the risk of adverse effects—a dynamic balance that reinforces the clinical importance of genotype-guided dosing (Bousman et al., 2023). Consistent with this, current Clinical Pharmacogenetics Implementation Consortium (CPIC) and Dutch Pharmacogenetics Working Group (DPWG) guidelines recommend a 50% dose reduction or the selection of alternative antidepressants for CYP2C19 PMs, and avoidance of predominantly CYP2C19- or CYP2D6-metabolized drugs in ultrarapid metabolizers to prevent therapeutic failure (Hicks et al., 2017).

## 8 Integrating pharmacogenetic evidence: CYP2D6 and CYP2C19 polymorphisms in antidepressant response and clinical translation

Although Li et al. (2024) reported no genome-wide significant correlations between metabolic phenotype and antidepressant response, their findings highlight key pharmacogenetic mechanisms linking CYP polymorphisms to drug pharmacodynamics via altered pharmacokinetics. Specifically, genetic variants in CYP2D6 and CYP2C19 determine individual variability in metabolic clearance and, consequently, plasma bioavailability of antidepressants, which in turn modulates synaptic serotonin reuptake inhibition and receptor occupancy—core pharmacodynamic determinants of antidepressant efficacy. The study thus reinforces the interplay between pharmacogenetic variability and clinical outcomes, advocating for the integration of genotype data into personalized therapeutic strategies (Li et al., 2024).

In the study by Zhiganova and Radkova (2025), the authors conclude that the observed difference in the prevalence of CYP2C19 genotypes (along with CYP2D6 and CYP1A2) in patients with resistance to antipsychotics and/or antidepressants enables the recommendation of pharmacogenetic testing for routine clinical practice. The objective is to select the most effective and safe treatment for these patients.

Moreover, Li et al. emphasize that future research combining genotype sequencing, pharmacokinetic monitoring, and comprehensive phenotyping (including adverse effects, co-medications, and environmental influences) will be critical to fully elucidate the contribution of CYP2D6 and CYP2C19 polymorphisms to individualized antidepressant therapy. This aligns with the broader concept that genetic influences on pharmacodynamics and pharmacokinetics are interdependent—where gene-driven differences in enzymatic metabolism directly alter drug exposure, influencing receptor interactions, signaling cascades, and ultimately clinical effectiveness (Li et al., 2024; Bousman et al., 2021).

Thus, the findings by Li et al. (2024) advance the understanding of genetic determinants of antidepressant pharmacokinetics, particularly emphasizing the complex impact of CYP2D6 polymorphisms. Despite methodological constraints, the study highlights the urgent need for clinically validated pharmacogenomic testing to personalize antidepressant dosing, mitigate adverse reactions, and optimize therapeutic efficacy across diverse populations.

Building upon the pharmacogenetic insights previously discussed by Li et al. (2024), the findings presented by Kee et al. (2023) further broaden the understanding of how CYP2D6 and CYP2C19 polymorphisms influence antidepressant pharmacokinetics, pharmacodynamics, and clinical response in real-world settings. Kee and colleagues conducted a detailed retrospective cohort analysis in New Zealand involving 52 mental-health patients who had experienced adverse drug reactions (ADRs) and/or ineffectiveness related to antidepressant treatments. By integrating Clinical Pharmacogenetics Implementation Consortium (CPIC) guidelines and confirmed genotyping data, the study sought to ascertain the degree to which pharmacogenetic variation explains variability in antidepressant efficacy and tolerability, ultimately providing empirical evidence for the clinical utility of personalized pharmacogenetic testing (Kee et al., 2023).

The study found that nearly 85% of individuals carried non-normal metabolizer (non-NM) phenotypes for CYP2D6 and/or CYP2C19—a distribution that aligns with data from European and other international cohorts (Maggo et al., 2019). Specifically, 57% of participants were CYP2D6 non-NMs and 62% were CYP2C19 non-NMs, mirroring earlier findings that most patients treated for depression harbor genotypes associated with altered enzymatic activity. This high prevalence underlines the clinical relevance of pharmacogenetic testing since deviations in metabolic capacity substantially modify antidepressant plasma concentrations and, consequently, pharmacodynamic outcomes (Kee et al., 2023). The authors noted that poor or intermediate metabolizers demonstrate reduced drug clearance, which can elevate systemic exposure and increase ADR risk, whereas rapid or ultrarapid metabolizers may experience subtherapeutic exposure and treatment failure. These patterns provide a pharmacokinetic explanation for interindividual differences in antidepressant effectiveness and tolerance, a theme consistent with prior large-scale investigations (Hicks et al., 2017; Li et al., 2024).

Importantly, Kee et al. (2023) distinguished between ADR-related and inefficacy-related outcomes. Among 79 CYP2D6/CYP2C19–antidepressant pairs with established CPIC evidence levels (A, A/B, or B), approximately 48% of ADR-associated pairs and 21% of ineffectiveness-associated pairs were actionable, signifying that genotype-guided interventions could potentially mitigate nearly half of ADR cases and one-fifth of therapeutic failures. These estimates echo those reported in larger clinical implementation trials such as the PREPARE study, which demonstrated reduced ADR rates when pharmacogenetic guidance informed prescribing decisions (Swen et al., 2023). Kee and colleagues emphasize that pharmacogenetics contributes more strongly to tolerability than to efficacy, a finding also supported by Mrazek et al. (2011), who found that CYP2C19 2 carriers had lower citalopram tolerability but no difference in remission rate.

The report recognizes the polygenic and multifactorial nature of antidepressant response, proposing that other pharmacodynamic genes (e.g., SLC6A4, HTR2A, ADRA2A) may interact with CYP-mediated pharmacokinetics to shape overall efficacy (Bahramali et al., 2016; van Westrhenen et al., 2020). Moreover, the authors highlighted phenoconversion—the modulation of metabolic activity by external factors such as drug-drug interactions—as a critical complicating variable. Empirical evidence indicates that individuals who are genetically normal metabolizers may phenotypically convert to poor metabolizers when co-administered with enzyme inhibitors, leading to unexpected ADRs even in the presence of benign genotypes (Cicali and Wiisanen, 2022; Gloor et al., 2022). Consequently, Kee et al. advocate for integrating pharmacogenetic data with comprehensive medication review to refine phenotype prediction and diagnostic precision in therapy management.

Current evidence strongly suggests the importance of aligning medication prescriptions with patients’ genetic indications (Serretti et al., 2025). Decision-support tools that incorporate dosage alerts based on guidelines such as those from CPIC are already being tested in primary care settings. A practical approach for implementation could include evaluating concomitant medications and the potential for phenoconversion; in cases where overexposure is anticipated (for example, in poor metabolizers for the primary pathway), considering a lower starting dose or slower titration with rigorous monitoring according to established guidelines; when underexposure is expected (for example, in ultrarapid metabolizers), cautiously increasing the dose within safety limits or selecting an alternative agent with less dependence on the affected metabolic pathway; and continuously monitoring efficacy and adverse effects (as assessed by the UKU), adjusting the treatment as necessary (Serretti et al., 2025).

From a clinical perspective, this study reinforces the translational value of CYP2D6 and CYP2C19 screening in psychiatry. Empirical evidence suggested that roughly 60% of antidepressant-related adverse or ineffective responses could be linked to actionable genotypes, corroborating the predictive validity of pharmacogenetic profiling. The study also connects genotype-dependent pharmacokinetic alteration to pharmacodynamic mechanisms: slower metabolizers possess higher plasma drug concentrations, leading to greater receptor occupancy and thus higher potential for both therapeutic and adverse effects, whereas ultrarapid metabolizers may fail to achieve sufficient receptor engagement, resulting in poor clinical response. This dynamic pharmacokinetic-pharmacodynamic interplay emphasizes why pharmacogenetic stratification can optimize dosage selection, improve remission rates, and reduce drug discontinuation due to intolerability (Arnone et al., 2023; Kee et al., 2023).

Kee and colleagues also addressed population heterogeneity, referencing previous studies that found variable allele frequencies across ethnic groups. For example, CYP2C19 2 and *3 alleles—associated with decreased metabolism—are notably frequent in Asian populations, while CYP2D6 10 is common in East Asians and Pacific Islanders (Hitchman et al., 2022). These findings imply that population-specific genetic architecture should be considered when applying universal pharmacogenetic guidelines, as recommended by the CPIC and DPWG (Caudle et al., 2020).

In terms of limitations, Kee et al. (2023) acknowledged the small sample size and partial clinical data, stressing the need for larger, prospective studies combining genotyping, serum drug-level monitoring, and clear phenotypic classifications of ADRs *versus* non-response. They also recommended expanding genotyping panels to include other relevant pharmacokinetic and pharmacodynamic genes beyond *CYP2D6* and *CYP2C19*, thereby capturing the full scope of genetic determinants influencing antidepressant response. The authors concluded that while pharmacogenetic information should not exclusively dictate prescribing decisions, it offers a valuable clinical framework to guide individualized treatment—enhancing the precision, safety, and effectiveness of antidepressant pharmacotherapy (Kee et al., 2023).

Overall, the complementary research of Kee et al. (2023) and Li et al. (2024) underscores the intricate relationship between genetic polymorphisms, pharmacokinetics, and pharmacodynamics. Together, both works demonstrate that *CYP2D6* and *CYP2C19* polymorphisms significantly influence antidepressant plasma exposure, which directly affects pharmacodynamic mechanisms underlying efficacy and side-effect profiles. Consequently, genotype-guided prescribing represents a pivotal step toward personalized psychiatry, where treatment optimization aligns with an individual’s metabolic capacity, reducing trial-and-error prescribing and contributing to more predictable therapeutic outcomes.

## 9 Relevant genes for SSRI action and antidepressant metabolism

Selective serotonin reuptake inhibitors (SSRIs), such as citalopram, fluoxetine, fluvoxamine, paroxetine, and sertraline, were developed as an alternative to tricyclic antidepressants (TCAs), with the aim of maintaining effective treatment for depression while improving safety and tolerability (Anderson, 2000). SSRIs exert their action by blocking the serotonin transporter (SERT) at presynaptic terminals, thereby preventing the reuptake of 5-HT. This results in increased synaptic availability of serotonin, prolonging its action on postsynaptic receptors and enhancing serotonergic neurotransmission (Chu and Wadhwa, 2023). In addition, SSRIs are considered the first-line pharmacological treatment for anxiety disorders, with pharmacogenetic response being influenced by polymorphisms in the *CYP2D6* and *CYP2C19* genes (Brouwer et al., 2022).

Although selective serotonin reuptake inhibitors (SSRIs) share a common mechanism of action centered on the blockade of serotonin reuptake transporters, they differ considerably in their chemical structures and secondary mechanisms, which contribute to variability in their pharmacodynamic and pharmacokinetic profiles. Among these agents, sertraline and paroxetine are recognized as two of the most potent SSRIs in terms of serotonin reuptake inhibition. Notably, sertraline also exhibits mild dopamine reuptake inhibition, which distinguishes its neurochemical and functional profile from other SSRIs, possibly contributing to its activating properties and clinical efficacy in certain depressive subtypes. Furthermore, SSRIs show differential interactions with other receptor systems, such as muscarinic, 5-HT_2_C, and sigma-1 receptors, which may influence both their therapeutic benefits and side effect spectra. Paroxetine, in particular, has been reported to exert inhibitory effects on nitric oxide synthase, an action that may underlie some of its anxiolytic and sedative properties. On the other hand, citalopram and fluoxetine consist of racemic mixtures containing enantiomers with distinct pharmacological activities—for example, the S-enantiomer of citalopram (escitalopram) exhibits a higher affinity and selectivity for the serotonin transporter compared to its R-form, leading to differences in potency and tolerability across clinical use (Sharp and Collins, 2024).

Most SSRIs are rapidly absorbed and exhibit high plasma protein binding (except for fluvoxamine and citalopram, which have lower affinity). Fluoxetine is unique among SSRIs in having an active metabolite and a long half-life, resulting in delayed onset of action and prolonged effects even after discontinuation. Conversely, SSRIs such as paroxetine and fluvoxamine demonstrate non-linear pharmacokinetics, meaning that increased doses do not lead to proportionate increases in plasma concentrations. This is due to dose-dependent inhibition of cytochrome P450 (CYP) enzymes (Preskorn, 1997; Zelek-Molik and Litwa, 2025).

Among these enzymes, the cytochrome P450 (CYP) superfamily—particularly CYP2D6 and CYP2C19—plays a central role in the hepatic metabolism of selective serotonin reuptake inhibitors (SSRIs) and in determining their pharmacokinetic variability among individuals. SSRIs remain the most widely prescribed antidepressants today due to their ability to selectively inhibit the serotonin transporter (SERT), thereby increasing extracellular serotonin levels and alleviating symptoms of major depression (Sharp and Collins, 2024).

Variations in CYP enzyme activity—whether due to genetic polymorphisms, co-administered drugs, or liver function—may therefore contribute to individual differences in both efficacy and adverse effects. These polymorphisms alter enzymatic activity, resulting in varying rates of drug clearance and bioavailability that contribute to both therapeutic variability and the risk of adverse reactions (Zelek-Molik and Litwa, 2025).

Clinically, SSRI-related side effects tend to involve multiple physiological systems. Gastrointestinal symptoms such as nausea, vomiting, abdominal discomfort, and diarrhea are among the most common, resulting from enhanced serotonergic activity in the enteric nervous system. Psychiatric and neurological reactions, including agitation, anxiety, akathisia, tremors, insomnia, and even mood elevation or manic switching in patients with bipolar disorder, have been documented. SSRIs may also produce sleep disturbances and fatigue, reflecting their variable influence on sleep architecture and energy regulation. Weight changes are variable and agent-dependent, with some patients experiencing mild weight loss early in therapy and others gaining weight during long-term use. Furthermore, sexual dysfunction—manifesting as decreased libido, delayed orgasm, or erectile difficulties—is a well-established class effect related to serotonergic modulation of spinal reflexes and cortical sexual drive pathways. In less common cases, dermatologic reactions such as rash or pruritus may occur, typically due to hypersensitivity or idiosyncratic immune responses (Chu and Wadhwa, 2023; Sepúlveda-Lizcano et al., 2023).

More broadly, the work of Sharp and Collins (2024) and Leichsenring et al. (2023) supports SSRIs as first-line antidepressant treatments in major depression under current clinical guidelines, while also acknowledging that delayed onset and variable outcomes persist due to underlying pharmacogenetic differences. Together, evidence from Li et al. (2024) and Kee et al. (2023) demonstrates that CYP polymorphisms substantially influence SSRI metabolism, shaping both pharmacokinetic exposure and clinical effectiveness. The genes CYP2D6 and CYP2C19 are thus considered key pharmacogenes governing SSRI action and serve as important biomarkers for guiding personalized antidepressant therapy.

## 10 Blood–brain barrier efflux transporters: the role of ABCB1 in antidepressant disposition

The pharmacokinetic variability of antidepressants is not solely determined by hepatic metabolism. In addition to cytochrome P450 enzymes, efflux transporters expressed at the blood–brain barrier (BBB) critically shape central nervous system (CNS) exposure to psychotropic drugs (O'Brien et al., 2012a; 2012b). The *ABCB1* gene encodes P-glycoprotein (P-gp), an ATP-dependent efflux pump highly expressed in brain capillary endothelial cells, which actively transports a wide range of xenobiotics from the brain interstitial space back into the systemic circulation (Chai et al., 2022; Skinner et al., 2023). Several antidepressants, including paroxetine, sertraline, venlafaxine and citalopram/escitalopram, have been reported as P-gp substrates to varying degrees, indicating that interindividual differences in *ABCB1* function may modulate both efficacy and tolerability of SSRI/SNRI therapy (O'Brien et al., 2012a; 2012b).

Among the most extensively studied *ABCB1* polymorphisms are rs1045642 (C3435T) and rs2032582 (G2677T/A), which have been associated in some cohorts with altered P-gp expression or transport capacity and with differential clinical response to antidepressants (Baba et al., 2021; Geers et al., 2022; Radosavljevic et al., 2023). Although effect sizes are generally modest and findings are not fully consistent across studies, these variants illustrate that BBB transport can influence CNS drug concentrations independently of systemic pharmacokinetics. Mechanistically, antidepressant disposition at the brain targets is determined by at least two sequential processes: (i) hepatic metabolism—mainly via CYP2C19 and CYP2D6—which governs total plasma concentrations, and (ii) P-gp–mediated efflux at the BBB, which defines the fraction of circulating drug that effectively reaches the CNS (O'Brien et al., 2012a; 2012b).

This layered pharmacokinetic model implies that *ABCB1* polymorphisms may attenuate or amplify the clinical impact of CYP2C19/CYP2D6 phenotypes (Ganoci et al., 2021; Islam et al., 2022; Yoo and Lee, 2011). For example, a patient who is a CYP2C19 poor metabolizer may exhibit elevated plasma escitalopram levels, yet reduced brain penetration if *ABCB1* variants confer increased P-gp activity (Brouwer et al., 2022; Huang et al., 2021; Sandritter et al., 2023); conversely, decreased transporter function could enhance CNS exposure even in normal metabolizers (Ding et al., 2025; Eyal et al., 2010). Therefore, future multigene predictive frameworks for antidepressant response should ideally integrate both metabolic (CYP2C19/CYP2D6) and transport (*ABCB1*) pathways when estimating therapeutic efficacy and adverse-effect risk.

## 11 Pharmacogenetic markers in depression treatment: the role of SLC6A4, HTR2A, CYP2D6, and CYP2C19

Depression is a condition of complex origin and multifactorial etiology, involving genetic, environmental, and biochemical components. Its pharmacological treatment is characterized by heterogeneous individual responses. Pharmacogenetics, which investigates how DNA variations influence drug response, has emerged as a strategic field in modern psychiatry (Table 1).

**TABLE 1:** Key pharmacogenetic determinants of SSRI response: pharmacokinetic and pharmacodynamic markers.

Gene	Variant/Phenotype	Functional effect	Main affected drugs (SSRIs/related)	Expected clinical impact	Suggested clinical action^a^
CYP2C19	*2, *3, other loss-of-function alleles → PM/IM	↓ CYP2C19 activity → ↓ clearance, ↑ plasma concentrations	Citalopram, escitalopram, sertraline	↑ efficacy probability but ↑ risk of dose-related adverse effects (QTc prolongation, GI symptoms, sedation)	Start at ∼50% of standard dose or choose alternative with less CYP2C19 dependence; use slow titration and consider TDM (CPIC 1A/1B)
CYP2C19	*17/*17 or *1/*17 → RM/UM	↑ CYP2C19 activity → ↑ clearance, ↓ plasma concentrations	Citalopram, escitalopram, sertraline	↓ likelihood of response due to subtherapeutic exposure	Consider higher dose within safety limits or switch to drug less dependent on CYP2C19; monitor clinical response and, if available, TDM.
CYP2D6	Null alleles (*3, *4, *5, *6, etc.) → PM / IM	↓ CYP2D6 activity → ↓ clearance of substrates, ↑ parent-drug levels, altered metabolite ratios	Paroxetine, fluoxetine, sertraline; venlafaxine; adjunctive aripiprazole	↑ risk of adverse effects (e.g., akathisia, mania, discontinuation) with CYP2D6 substrates; for venlafaxine, ↓ formation of active metabolite	Prefer non-CYP2D6-dependent agents or reduce starting dose; avoid high doses of venlafaxine and aripiprazole; monitor tolerability closely
CYP2D6	Gene duplication/increased-function alleles → UM	↑ CYP2D6 activity → ↑ clearance, ↓ exposure to parent drug and active metabolites	Paroxetine, fluoxetine, sertraline; venlafaxine	Possible non-response or partial response due to underexposure	Use drugs with less CYP2D6 dependence or consider dose escalation within guideline limits, with close monitoring of efficacy
SLC6A4	5-HTTLPR S/S	↓ SERT expression → reduced reuptake capacity, altered SSRI pharmacodynamics	Class effect for SSRIs	↓ probability of response and remission; ↑ risk of adverse effects, especially under high environmental stress	Consider closer monitoring, slower titration, and earlier switch strategy if no response; combine with CYP information in algorithms
SLC6A4	5-HTTLPR L/L	↑ SERT expression → greater dynamic range for SSRI inhibition	Class effect for SSRIs	↑ response and remission rates, particularly in European cohorts	Standard SSRI dosing; genotype may support persistence before declaring non-response
HTR2A	rs7997012 A allele; rs6313 T allele	Modulates 5-HT2A receptor expression/signaling, influencing SSRI tolerability and response	Class effect for SSRIs	Small/moderate effects on response and side-effect profile; evidence less consistent than for CYP2C19/CYP2D6	Use as secondary modifier in multigene panels rather than as standalone marker; not yet guideline-mandated for dosing decisions

↓ Indicates reduced activity; ↑ indicates increased activity. PM, poor metabolizer; IM, intermediate metabolizer; NM, normal metabolizer; RM, rapid metabolizer; UM, ultrarapid metabolizer; SERT, serotonin transporter; TDM, therapeutic drug monitoring.

^a^ Actions summarise current tendencies in CPIC/DPWG, and related guidelines; they should always be integrated with clinical judgement, comorbidities, and concomitant medications.

**FIGURE 2:**
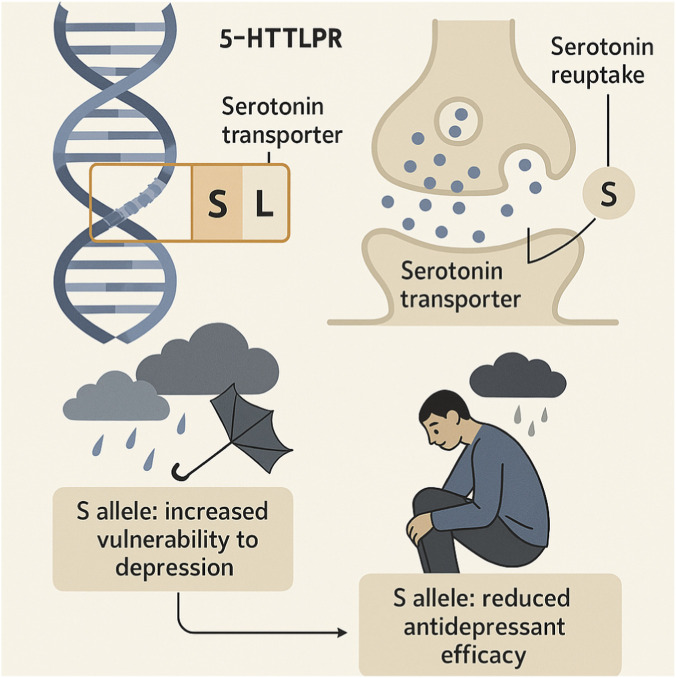
Infographic illustrating the effects of polymorphisms in the serotonin transporter gene (SLC6A4), with a focus on the 5-HTTLPR variant. The presence of the “S” allele is associated with reduced serotonin reuptake, increased vulnerability to depression, and diminished therapeutic response to antidepressants, particularly under environmental stress. The image integrates genetic, neurochemical, and environmental elements to highlight the multifactorial nature of depressive disorders.

Building upon this pharmacogenetic framework, the serotonin transporter gene (*SLC6A4*), which encodes the target of selective serotonin reuptake inhibitors (SSRIs), represents one of the most extensively investigated genetic determinants of antidepressant response. Variants within this gene, especially the 5-HTTLPR (serotonin-transporter-linked polymorphic region) located in the promoter region, exert significant influence over serotonin transporter expression and subsequent synaptic serotonin availability (Canli and Lesch, 2007). The short (“S”) allele of 5-HTTLPR is associated with reduced transcriptional efficiency, leading to lower transporter density on presynaptic membranes and consequently diminished serotonin reuptake capacity (Karg et al., 2011). Functionally, this polymorphism modulates both the *efficacy* and *safety* of SSRI therapy: individuals carrying the S allele tend to exhibit blunted antidepressant response, slower onset of remission, and increased susceptibility to adverse effects, particularly when exposed to stressful environmental factors that intensify serotonergic dysregulation (Figure 2) (Caspi et al., 2003; Castro et al., 2024).

In pharmacodynamic terms, 5-HTTLPR variation alters the neural homeostasis that underlies treatment response. The transporter encoded by *SLC6A4* serves as the primary site of action for SSRIs—such as fluoxetine, sertraline, and escitalopram—which inhibit serotonin reuptake to enhance synaptic availability (Sharp and Collins, 2024). When this transporter is underexpressed due to the S allele, SSRIs face a functional ceiling effect—their capacity to further elevate extracellular serotonin is reduced, leading to incomplete receptor stimulation and, therefore, suboptimal antidepressant efficacy (Porcelli et al., 2012). Conversely, carriers of the long (“L”) allele maintain higher transporter expression, enabling a greater dynamic range of SSRI inhibition and more favorable treatment outcomes. Notably, meta-analyses confirm that 5-HTTLPR exerts a gene × environment (G × E) interaction effect: the depressive risk and treatment results depend not solely on genotype but on the interplay between genetic predisposition and environmental adversity (Uher and McGuffin, 2010).

Furthermore, current pharmacogenetic models suggest that the combined influence of pharmacokinetic genes (e.g., *CYP2D6* and *CYP2C19*) and pharmacodynamic genes (e.g., *SLC6A4*, *HTR2A*, *BDNF*) collectively shape antidepressant response (Li et al., 2024; Kee et al., 2023). While cytochrome P450 enzymes modulate plasma drug levels and bioavailability, the serotonin transporter directly determines synaptic pharmacodynamics—together contributing to the interindividual variability observed in SSRI efficacy and tolerability. For instance, a patient genetically predisposed to low CYP2C19 activity (poor metabolizer phenotype) and carrying the 5-HTTLPR *S* allele may experience higher systemic drug exposure yet diminished pharmacodynamic response at the receptor level, underscoring the multifactorial nature of antidepressant outcomes (Table 2) (Bousman et al., 2023).

**TABLE 2:** Summary of key pharmacogenetic recommendations and indicative strength of evidence.

Gene/marker	Genotype/phenotype	Clinical recommendation	Indicative evidence grade^a^
CYP2C19	Poor metabolizer (e.g., *2/*2, *2/*3, other loss-of-function alleles)	For citalopram and escitalopram, reduce starting dose by ∼50% or select an alternative antidepressant with less CYP2C19 dependence; monitor closely for dose-related adverse effects and consider TDM when available	Level 1A – Strong, guideline-supported dose/drug-selection recommendation (CPIC/DPWG SSRIs; multiple clinical studies)
CYP2C19	Ultrarapid metabolizer (e.g., *17/*17)	Avoid predominantly CYP2C19-metabolized antidepressants or consider higher doses within approved ranges; preferentially use agents with reduced CYP2C19 dependence if there is inadequate response at standard doses	Level 1A – Strong, guideline-supported recommendation to avoid or adjust dose (CPIC/DPWG SSRIs/TCAs; meta-analyses)
CYP2D6	Poor/intermediate metabolizer (e.g., *3, *4, *5, *6, other null/decreased-function alleles)	For paroxetine, fluoxetine and other CYP2D6-substrate antidepressants and adjunctive aripiprazole, use reduced starting doses or select alternatives; avoid high venlafaxine doses in PMs because of altered parent/metabolite balance; monitor tolerability and consider TDM.	Level 1A – Strong, guideline-supported dose adjustment/drug-selection recommendation (CPIC/DPWG; implementation studies)
CYP2D6	Ultrarapid metabolizer (gene duplication or increased-function alleles)	Avoid paroxetine, fluoxetine and venlafaxine as first-line options, or consider dose escalation within safety limits if used; prefer drugs with minimal CYP2D6 dependence when prior non-response is documented	Level 1A/1B – Guideline-supported recommendation to avoid or up-titrate (CPIC/DPWG; moderate clinical trial evidence)
SLC6A4 (5-HTTLPR)	S/S genotype (short/short)	Consider this variant as a secondary modulator of SSRI response and tolerability: Anticipate lower probability of remission and higher risk of adverse effects, particularly under high stress; use closer monitoring, slower titration, and a lower threshold for switching if inadequate improvement	Level 2B/3 – Emerging evidence; information-modifying marker (multiple meta-analyses, but no formal dosing guideline)
SLC6A4 (5-HTTLPR)	L/L genotype (long/long)	Recognize a modestly higher likelihood of response and remission to SSRIs, particularly in European cohorts; standard dosing applies, with genotype used mainly to support persistence before declaring non-response	Level 2B – Moderate, consistent association with response but not guideline-mandated
HTR2A	rs7997012 (A allele carriers vs. CC); rs6313 (T vs. C)	May be considered as secondary modifiers of SSRI response and side-effect profile; can inform expectations about efficacy/tolerability in multigene panels, but should not be used alone to dictate drug choice or dosing	Level 2B/3 – Suggestive association; exploratory/complementary information (meta-analyses; CPIC does not recommend dosing changes)
ABCB1	rs1045642, rs2032582 and related variants	May influence blood–brain barrier efflux of several SSRIs/SNRIs and thus CNS exposure; currently best viewed as a research marker to explain variability rather than as a routine clinical determinant of dose or drug choice	Level 3 – Experimental/hypothesis-generating marker (heterogeneous and non-standardized evidence)

^a^ Evidence grades are indicative and based on convergence between CPIC/DPWG, guideline status for CYP2C19/CYP2D6 and the broader pharmacogenetic literature for SLC6A4, HTR2A and ABCB1. They are intended to distinguish mandatory, guideline-level actions (e.g., dose reduction or drug avoidance) from optional, information-modifying markers, rather than to reproduce an official CPIC, grading system.

Hence, as Castro et al. (2024) emphasize, variations in the *SLC6A4* gene—particularly the 5-HTTLPR polymorphism—are critical in elucidating the broad heterogeneity in antidepressant efficacy and safety. The presence of the S allele, associated with reduced transporter expression and amplified stress sensitivity, exemplifies how genetic factors interact with environmental triggers to influence vulnerability to depression and treatment success. This integrative perspective aligns with contemporary pharmacogenomic paradigms advocating for multigene and environment-informed models to achieve truly personalized antidepressant therapy.

Another critical aspect involves drug-metabolizing enzymes such as *CYP2C19*. Genetic variants in this gene result in different metabolic phenotypes. Ultrarapid metabolizers tend to show higher scores on the Hamilton Depression Rating Scale (HAM-D), suggesting insufficient clinical response, whereas poor metabolizers are at increased risk of toxicity due to drug accumulation. These findings underscore the relevance of pharmacogenetics in individualizing drug dosage (Hoefel, 2023; Li et al., 2024).

Thus, there is a need to deepen our understanding of the impact of genetic polymorphisms—both in the serotonergic system and hepatic metabolism—on antidepressant treatment response. Integrating such genetic markers into clinical practice may lead to more accurate therapy selection, reduced adverse effects, and more efficient use of healthcare resources, thereby establishing pharmacogenetics as a key element in precision medicine. To enhance clinical applicability and move beyond single-gene descriptions, illustrative multigene scenarios can be used to demonstrate how combined pharmacokinetic (CYP2D6, CYP2C19) and pharmacodynamic (SLC6A4, HTR2A) information may guide antidepressant selection, dosing, and monitoring. Table 3 summarizes selected genotype constellations and proposes individualized prescribing strategies that align with current CPIC and DPWG recommendations while highlighting areas where evidence remains emerging.

**TABLE 3:** Illustrative genotype combinations and suggested personalized antidepressant strategies.

Genotype combination	Expected pharmacokinetic/pharmacodynamic effect	Preferred drug options	Dose and titration suggestions	Monitoring and clinical notes
CYP2C19 poor metabolizer (PM) + SLC6A4 5-HTTLPR S/S	↓ CYP2C19 activity → ↑ plasma levels of CYP2C19-substrate SSRIs; 5-HTTLPR S/S → ↓ SERT expression and ↓ SSRI pharmacodynamic response	Prefer agents with minimal CYP2C19 dependence: desvenlafaxine, mirtazapine, agomelatine. If SSRI needed: sertraline > escitalopram/citalopram	If escitalopram or citalopram are used: start at ∼50% of standard starting dose (e.g., escitalopram 5 mg/day), with slow titration as tolerated	Close monitoring for dose-related AEs (GI symptoms, sedation, QTc). Given S/S genotype and higher toxicity risk, reassess efficacy early (4–6 weeks) and switch class if needed
CYP2D6 ultrarapid metabolizer (UM) + HTR2A rs7997012 CC	↑ CYP2D6 activity → ↑ clearance and ↓ exposure to CYP2D6-substrate antidepressants; HTR2A CC → modestly ↓ SSRI response in some cohorts	Avoid CYP2D6-dependent first-line choices (paroxetine, fluoxetine, venlafaxine, adjunctive aripiprazole). Prefer escitalopram, sertraline, or non-SSRIs (desvenlafaxine)	If venlafaxine is chosen, consider higher doses within guideline limits (e.g., 225–300 mg/day) with gradual titration and BP/HR monitoring	High risk of underexposure and partial response; use structured scales (e.g., HAM-D, PHQ-9) at 4–6 weeks; adjust dose or switch early if response <25%
CYP2C19 PM + CYP2D6 PM	Markedly ↓ clearance of several SSRIs/TCAs → ↑ plasma concentrations and high risk of adverse effects and discontinuation	Prefer drugs with limited CYP2C19/CYP2D6 metabolism (e.g., desvenlafaxine, agomelatine, vortioxetine, mirtazapine)	If a CYP-substrate SSRI is used, start at 25%–50% of standard dose and titrate very slowly; consider pre-emptive TDM where available	Strong indication for genotype-guided prescribing; monitor for sedation, QTc prolongation, hyponatremia, and switch strategy if intolerance persists
CYP2C19 RM/UM (*17 carriers) + SLC6A4 L/L	↑ CYP2C19 activity → ↓ exposure to CYP2C19-substrate SSRIs; 5-HTTLPR L/L → ↑ SERT expression and potentially better SSRI responsiveness	SSRIs still appropriate, but consider sertraline or non-CYP2C19-dominant drugs if citalopram/escitalopram fail	For citalopram/escitalopram, consider faster titration up to upper end of recommended dose range if tolerability is good	Emphasis on efficacy rather than toxicity: if no remission after adequate high-range dosing and 6–8 weeks, switch to alternative mechanism (e.g., SNRI)
CYP2D6 IM/PM + use of adjunctive aripiprazole	↓ CYP2D6 activity → ↑ aripiprazole levels and risk of akathisia, extrapyramidal symptoms, and discontinuation	If augmentation is needed, consider lower-dose aripiprazole or alternative augmenting strategies (e.g., lithium, quetiapine) according to guidelines	Start aripiprazole at half the usual starting dose and titrate slowly (e.g., 1–2 mg/day in sensitive patients), guided by tolerability	Monitor for akathisia, agitation, insomnia; early dose reduction or discontinuation can prevent treatment failure due to intolerance

↓ Indicates reduced activity; ↑ indicates increased activity. PM, poor metabolizer; IM, intermediate metabolizer; NM, normal metabolizer; RM, rapid metabolizer; UM, ultrarapid metabolizer; SERT, serotonin transporter; TDM, therapeutic drug monitoring; AE, adverse event.

The variability in clinical response to selective serotonin reuptake inhibitors (SSRIs) remains a recurrent challenge in the treatment of depressive disorders. Multiple studies have demonstrated that this heterogeneity is partly attributable to genetic factors that influence both the pharmacokinetics and pharmacodynamics of these agents. Among the most studied genes in this context are *SLC6A4*, *HTR2A*, *CYP2D6*, and *CYP2C19*, whose variants have significant implications for the efficacy and safety of SSRIs (Porcelli et al., 2012; Hicks et al., 2015).

From a pharmacodynamic perspective, the *SLC6A4* gene, which encodes the serotonin transporter (5-HTT), is one of the most extensively studied in antidepressant pharmacogenetics. The key functional variant, a short/long repeat polymorphism (5-HTTLPR), influences the expression level of the transporter. The short (S) allele is associated with reduced transcription and, consequently, with lower clinical efficacy of SSRIs and a higher incidence of adverse effects, especially in individuals with the S/S genotype (Kato and Serretti, 2010; Serretti et al., 2007). Meta-analyses indicate that the long (L) allele is associated with better treatment response in European populations (Porcelli et al., 2012).

Another pharmacodynamic gene of interest is *HTR2A*, which encodes the 5-HT2A serotonin receptor. Polymorphisms such as rs7997012 and rs6313 have been associated, in some cohorts, with more favorable responses to SSRIs and reduced occurrence of side effects (McMahon et al., 2006; Niitsu et al., 2013). However, the consistency of these associations remains limited, and clinical guidelines—such as those issued by the Clinical Pharmacogenetics Implementation Consortium (CPIC)—do not currently recommend the use of *HTR2A* as a standalone clinical biomarker (Hicks et al., 2015).

**FIGURE 3:**
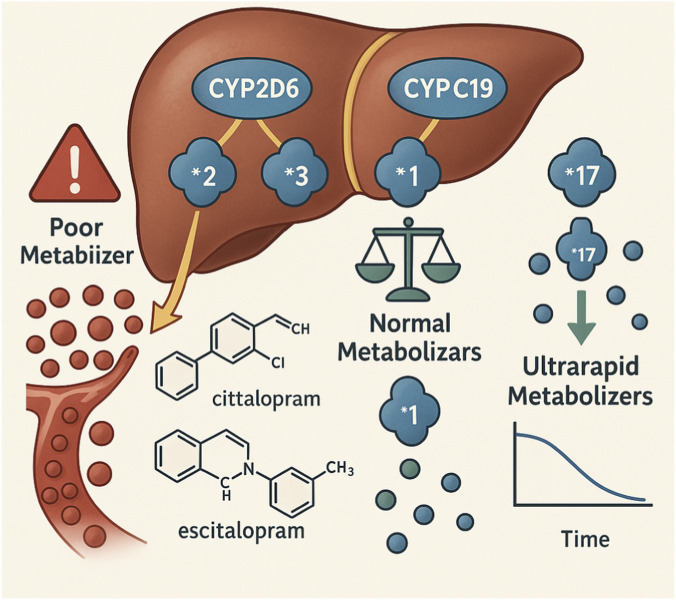
Illustration of the liver highlighting the roles of CYP2D6 and CYP2C19 enzymes in the metabolism of SSRI antidepressants, such as citalopram and escitalopram. The image depicts the effects of genetic polymorphisms (*2, *3, *17) on enzymatic activity, categorizing individuals as poor metabolizers (drug accumulation in the bloodstream with risk of toxicity), normal metabolizers (balanced metabolism and therapeutic effect), and ultrarapid metabolizers (accelerated drug clearance and potential treatment inefficacy). It includes icons representing chemical structures, genetic phenotypes, and pharmacokinetic graphs.

Regarding pharmacokinetics, the *CYP2D6* and *CYP2C19* genes, both part of the cytochrome P450 enzyme family, play a central role in the hepatic metabolism of SSRIs. Variants such as *CYP2C19* *2, *3, and *17 are associated with distinct metabolic profiles, directly impacting plasma concentrations of drugs such as citalopram and escitalopram (Faraj et al., 2024; Hicks et al., 2015). Poor metabolizers (e.g., *2/*2 or *2/*3 genotypes) are at increased risk of toxicity, while ultrarapid metabolizers (e.g., *17/*17) may fail to reach therapeutic drug levels, necessitating dose adjustments or drug substitution (Figure 3).

Similarly, *CYP2D6* is crucial for the metabolism of paroxetine, fluoxetine, and sertraline. Genotypes associated with reduced or null enzymatic activity (e.g., *3, *4, *5, *6) result in drug accumulation and an elevated risk of adverse effects, whereas ultrarapid metabolizers may show suboptimal therapeutic responses (Faraj et al., 2024; Hicks et al., 2015). CPIC and the Dutch Pharmacogenetics Working Group (DPWG) have already issued clinical guidelines with SSRI selection and dosing recommendations based on individual *CYP2D6* and *CYP2C19* metabolizer profiles (Hicks et al., 2015).

## 12 Towards individualized antidepressant regimens: illustrative genotype–based scenarios

Translating pharmacogenetic findings into clinical decision-making requires moving beyond single-gene associations and considering the combined impact of pharmacokinetic and pharmacodynamic variation (Pirmohamed, 2024; Sadee et al., 2023). Below, we present illustrative clinical scenarios that exemplify how multigene profiles can inform antidepressant selection and dosing. These examples are not meant as prescriptive algorithms, but as prototypes demonstrating how CYP2C19/CYP2D6 activity can be integrated with serotonergic markers such as *SLC6A4* and *HTR2A* in personalized regimens.

Scenario 1 – CYP2C19 poor metabolizer with SLC6A4 S/S genotype:

A patient with a CYP2C19 poor metabolizer phenotype (e.g., *CYP2C19* *2/*2) and the 5-HTTLPR S/S genotype is expected to exhibit reduced metabolic clearance of CYP2C19-dependent SSRIs and, simultaneously, diminished pharmacodynamic responsiveness to serotonin reuptake inhibition (Campos et al., 2022; Wong et al., 2023). For such individuals, antidepressants with limited reliance on CYP2C19, such as desvenlafaxine, agomelatine, or mirtazapine, may be preferred to avoid excessive plasma exposure and dose-related toxicity (Bousman et al., 2023; Joković et al., 2022). If an SSRI is clinically indicated—for example, due to comorbid anxiety or prior response—sertraline, which is metabolized by multiple pathways and is less dependent on CYP2C19, may be safer than citalopram or escitalopram (Kendrick et al., 2019; Poweleit et al., 2023). In line with CPIC/DPWG guidance, when citalopram or escitalopram are nonetheless prescribed, therapy should begin at approximately 50% of the standard starting dose (e.g., escitalopram 5 mg/day instead of 10 mg/day), followed by slow titration with careful monitoring for adverse effects such as gastrointestinal intolerance, sedation, and QTc prolongation (Brouwer et al., 2022). The concurrent S/S genotype suggests a higher likelihood of suboptimal efficacy and intolerance, underscoring the importance of early reassessment of response and a low threshold for switching to an alternative class if remission is not achieved.

Scenario 2 – CYP2D6 ultrarapid metabolizer with HTR2A rs7997012 CC genotype:

A second example involves a patient classified as a CYP2D6 ultrarapid metabolizer (UM) due to gene duplication or increased-function alleles, combined with the *HTR2A* rs7997012 CC genotype (García-González et al., 2023), which has been associated in some cohorts with less favorable SSRI outcomes (Bousman et al., 2023; Nahid and Johnson, 2022). In this context, CYP2D6 substrates such as paroxetine, fluoxetine, venlafaxine, and adjunctive aripiprazole are at increased risk of rapid clearance and underexposure, predisposing to non-response despite adequate dosing (Nahid and Johnson, 2022). As a result, first-line options might prioritize antidepressants that are less dependent on CYP2D6 metabolism, such as escitalopram or sertraline (with careful consideration of CYP2C19 status), or non-SSRI agents like desvenlafaxine (Calleja et al., 2023). If venlafaxine is chosen—for example, in patients with prominent comorbid anxiety or partial response history—higher-than-usual doses within guideline limits (e.g., 225–300 mg/day) may be required to achieve therapeutic concentrations of the active metabolite desvenlafaxine, while closely monitoring blood pressure, heart rate, and noradrenergic adverse effects (Lense et al., 2024). The rs7997012 CC genotype further suggests that pharmacodynamic sensitivity at the 5-HT2A receptor may be attenuated, reinforcing the need for rigorous outcome monitoring (e.g., systematic rating scales at 4–6 weeks) and timely adjustment of treatment if meaningful symptom reduction is not observed (Del Casale et al., 2025).

These scenarios illustrate how multigene profiles can refine initial drug choice (e.g., avoiding primary CYP-substrates in extremes of metabolism), starting dose, titration speed, and monitoring intensity. In clinical practice, such strategies should always be integrated with comorbidities, concomitant medications, and patient preferences, and interpreted in the context of evidence-based guidelines such as those from CPIC and DPWG.

## 13 Considerations for special populations: pediatrics, geriatrics, and clinical comorbidities

The translation of pharmacogenetic findings into clinical practice must account for the distinct physiological and pharmacological profiles of special populations, where age, organ function, and polypharmacy can significantly modulate genotype–phenotype relationships (Principi et al., 2023).

Enzyme ontogeny critically influences drug metabolism in children. While CYP2D6 activity reaches adult levels within the first year of life, CYP2C19 and CYP3A4 mature more slowly, leading to age-dependent variability in metabolic capacity (Subash and Prasad, 2024). Consequently, the probability of phenoconversion—where a patient’s metabolic phenotype is altered by drug–drug interactions or developmental changes—is particularly high in this population. For example, a child genotyped as a CYP2C19 normal metabolizer may exhibit a poor-metabolizer phenotype if co-administered a strong inhibitor (Cicali and Wiisanen, 2022). Therefore, even more conservative starting doses are recommended for children predicted to be poor or intermediate metabolizers based on genotype, accompanied by very close clinical and, when available, therapeutic drug monitoring (TDM) to avoid toxicity and ensure efficacy.

Older adults present a distinct challenge due to age-related decline in hepatic and renal function, increased body fat-to-lean mass ratio, and a high prevalence of polypharmacy (Ngcobo, 2025; Sutanto, 2025). In this context, the functional impact of genetic polymorphisms can be amplified: an intermediate metabolizer (IM) may effectively behave as a poor metabolizer (PM) because of reduced organ reserve. Dose adjustments are almost always necessary, and the choice of antidepressant should prioritize agents with a lower potential for drug–drug interactions and a favorable safety profile in the elderly. For instance, escitalopram (which has a relatively clean interaction profile) is often preferred over fluoxetine or paroxetine (potent CYP2D6 inhibitors) to minimize the risk of adverse events and complex pharmacokinetic interactions (van Poelgeest et al., 2021).

Systemic illnesses such as hepatic cirrhosis or severe renal impairment can mask the genetically predicted phenotype (Raghavan et al., 2026). In advanced liver disease, for example, the overall metabolic capacity may be so profoundly reduced that the contribution of a specific CYP polymorphism becomes negligible; genotyping alone loses its predictive value (Palatini and De Martin, 2016). Under these conditions, therapeutic drug monitoring (TDM) becomes mandatory to guide dosing, as plasma drug concentrations reflect the net effect of genetics, organ function, and concomitant medications. Similarly, in patients with significant cardiac, renal, or endocrine comorbidities, TDM should be considered an essential adjunct to genotype-guided prescribing to individualize therapy safely (Kehinde et al., 2023; Liang et al., 2024).

## 14 Alignment with clinical guidelines and addressing current controversies

The translation of pharmacogenetic findings into routine practice is guided by recommendations from regulatory agencies and international consortia, which sometimes adopt distinct stances (Omran et al., 2026; Pritchard et al., 2022). The U.S. Food and Drug Administration (FDA) includes pharmacogenetic information in drug labeling—for instance, issuing warnings about CYP2C19 poor metabolizers and the risk of QT-interval prolongation with citalopram—but does not mandate pre-emptive testing, maintaining a position of enforcement discretion (Drozda et al., 2018). In contrast, consortia such as the Clinical Pharmacogenetics Implementation Consortium (CPIC) and the Dutch Pharmacogenetics Working Group (DPWG) publish detailed, evidence-based guidelines that recommend specific actions (e.g., dose adjustments or alternative drug selection) for defined genotype-phenotype groups (Beunk et al., 2024; Díaz-Villamarín et al., 2025).

The core of the ongoing controversy revolves around two major points. First, critics emphasize the lack of large-scale, randomized controlled trials (RCTs) demonstrating that routine genotyping improves primary clinical outcomes—such as remission rates or time to recovery—in a cost-effective manner for the broad population of depressed patients (Baum et al., 2024; Brown et al., 2022). Second, the complexity of gene-gene-environment (GxGxE) interactions makes it difficult to predict treatment response based on a limited set of single-nucleotide polymorphisms (SNPs). Consequently, the greatest practical utility of current pharmacogenetic testing may lie more in preventing severe adverse drug reactions (Level 1A/B evidence, e.g., for CYP2C19/CYP2D6) than in accurately predicting efficacy (Level 2A/B evidence, e.g., for SLC6A4 or HTR2A) (Chenchula et al., 2024; Roberts et al., 2023).

In this context, the model proposed in this review seeks a balanced perspective. We acknowledge that guideline recommendations are more robust for pharmacokinetic (CYP) genes—where the relationship between genotype, drug concentration, and clinical outcome is well-established—than for pharmacodynamic markers (SLC6A4, HTR2A), where evidence is often derived from association studies and meta-analyses (Dhieb and Bastaki, 2025). Therefore, we posit that pharmacogenetic data should be viewed as one decisive piece of the clinical puzzle, to be integrated with a thorough patient history, assessment of comorbidities, and—when available—therapeutic drug monitoring (TDM). This approach is particularly valuable in cases of treatment resistance or a history of intolerance, where genotype-guided decisions can help optimize safety and increase the likelihood of a successful therapeutic outcome (Mhandire and Goey, 2022; Peña-Martín et al., 2022).

## 15 The impact of CYP2D6, CYP2C19, and serotonergic gene variants on antidepressant treatment outcomes

Despite numerous studies associating CYP2D6 and CYP2C19 isoenzyme genotypes with the metabolism of tricyclic antidepressants and selective serotonin reuptake inhibitors (SSRIs), clinical outcomes remain inconsistent. This is largely due to the difficulty in controlling confounding variables such as age, sex, psychiatric comorbidities, and the concomitant use of other medications, which limits the reproducibility and generalizability of findings (Frye and Nemeroff, 2024).

As SSRIs represent the primary class of antidepressants currently in use, the main biological markers studied to date are components of the serotonergic neurotransmission system, including synthetic enzymes, transporters, receptors, and metabolic enzymes for this neurotransmitter (Veenstra-VanderWeele et al., 2000).

**FIGURE 4:**
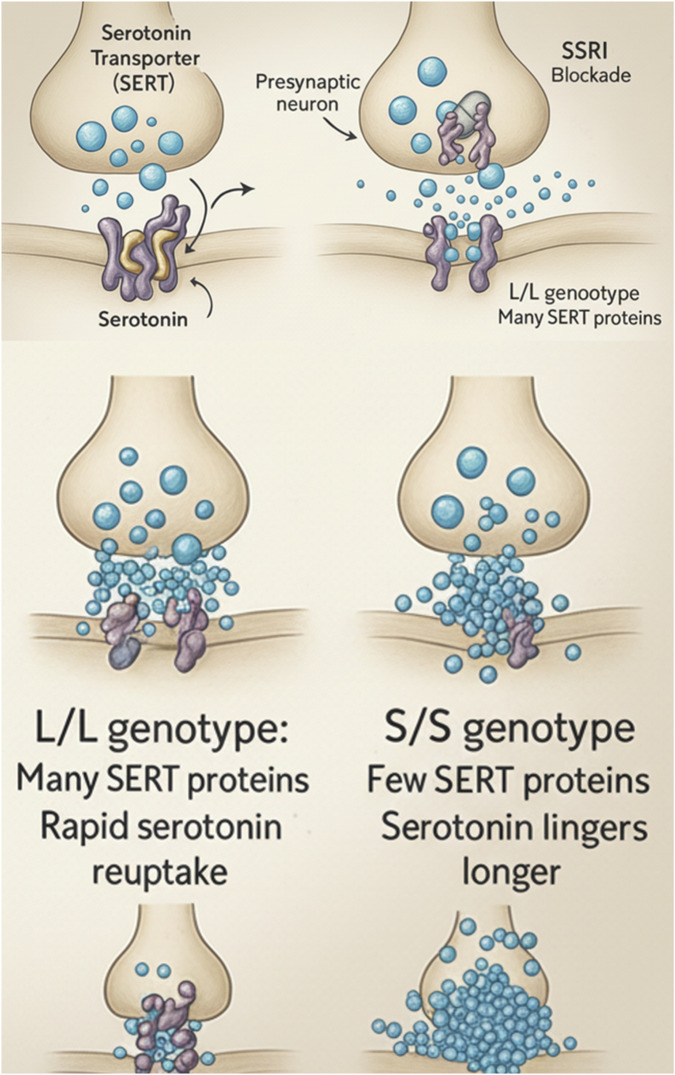
Infographic which illustrates the influence of polymorphisms in the serotonin transporter gene (SLC6A4), particularly the 5-HTTLPR (serotonin-transporter-linked polymorphic region) variant, on antidepressant response and depression vulnerability. The presence of the short (“S”) allele is associated with reduced serotonin (5-HT) reuptake, increased susceptibility to depressive disorders, and diminished efficacy of selective serotonin reuptake inhibitors (SSRIs), especially under environmental stress. The figure integrates genetic, neurochemical, and environmental components to emphasize the multifactorial nature of depression. SLC6A4: Solute Carrier Family 6 Member 4 (serotonin transporter gene); 5-HTTLPR: Serotonin-Transporter-Linked Polymorphic Region; 5-HT: 5-Hydroxytryptamine (serotonin); and SSRIs: Selective Serotonin Reuptake Inhibitors.

SSRIs are known to interfere with the activity of the serotonin transporter protein, which is responsible for removing serotonin from the synaptic cleft (Figure 4). Consequently, variants in the serotonin transporter gene *5HTT* have been extensively investigated. Lesch et al. (1996) and Heils et al. (1996) described a polymorphism in the promoter region of the *5HTT* gene that consists of a 44-base pair insertion or deletion, resulting in a long (L) and a short (S) allele. The L allele is associated with approximately twice the transcriptional activity of the S allele (Bousman et al., 2023; Li et al., 2024).

Since then, several studies have demonstrated an association between the S allele and poor response to SSRI treatment.

Integrating this genetic information into clinical algorithms allows for optimization of antidepressant selection and initial dosing, contributing to a reduction in adverse events and increasing the likelihood of therapeutic response. Although *CYP2C19* and *CYP2D6* currently have greater clinical applicability, there remains a need to systematize and consolidate the accumulated evidence regarding *SLC6A4* and *HTR2A*, particularly through meta-analyses stratified by ethnicity and sex, to enhance their clinical predictive value (Li et al., 2024; Kee et al., 2023).

The article also noted that pharmacogenomic support may be particularly advantageous for individuals who have failed multiple antidepressant regimens and for those with moderate to severe depression. In this systematic review, the clinical studies analyzed included testing for the *CYP2D6* and *CYP2C19* genes. However, commercial pharmacogenomic tests also include genes such as *SLC6A4* (serotonin transporter gene) and *HTR2A* (serotonin receptor gene). These pharmacodynamic genes have limited evidence supporting a direct effect on antidepressant efficacy (Brown et al., 2022).

## 16 Translation to clinical practice: integrating genotyping and therapeutic drug monitoring (TDM)

The clinical utility of pharmacogenetics depends on its ability to generate actionable recommendations (Adiukwu et al., 2023). Genotyping, whether performed pre-emptively or at the time of prescribing, provides a static prediction of the metabolic profile (Tortora, 2023); however, the final phenotypic expression may be modified by factors such as drug–drug interactions (phenoconversion), comorbidities and individual variability (de Jong et al., 2023). Integrating pharmacogenetic data with Therapeutic Drug Monitoring (TDM) is therefore essential to operationalize pharmacogenetic findings, allowing real-time treatment adjustments based on the patient’s actual exposure to the medication (Björkman, 2024; Ingelman-Sundberg and Molden, 2025).

For antidepressants with a defined therapeutic window, such as SSRIs, TDM offers objective phenotypic markers that complement genotype-based information (Bertollo et al., 2025; Parshenkov et al., 2025; Piacentino et al., 2022). In poor metabolizers, elevated plasma levels of the parent drug are expected; a pragmatic criterion for dose reduction is a plasma concentration consistently above the 75th percentile of the established therapeutic range or the emergence of clearly dose-dependent adverse effects (for example, marked nausea, sedation or dizziness) within the first two weeks of treatment at a standard starting dose (Celestin and Musteata, 2021). In ultrarapid metabolizers, in contrast, low plasma levels of the parent drug are frequently observed, often accompanied by a high active-metabolite-to-parent ratio; in this context, underdosing should be suspected when there is therapeutic failure—defined as less than a 25% reduction in scores on rating scales such as the HAM-D or MADRS after four to 6 weeks of treatment at a standard therapeutic dose—together with plasma concentrations persistently in the lower half or below the therapeutic range (Dean and Kane, 2012).

Although our discussion primarily focuses on CYP2D6 and CYP2C19, CYP3A4 also contributes to the metabolism of several SSRIs, including escitalopram (Islam et al., 2022; Jang et al., 2025). Consequently, strong CYP3A4 inhibitors (e.g., certain azole antifungals, macrolides) or inducers (e.g., carbamazepine, rifampicin, chronic smoking) may further shift the effective metabolic phenotype and plasma exposure, particularly in patients who are already genetically predisposed to reduced or increased metabolic capacity (Wang et al., 2022). When such co-medications are present, careful review of potential interactions and, where feasible, TDM are recommended to prevent unexpected toxicity or therapeutic failure.

To optimize safety and efficacy, a two-step monitoring strategy can be recommended (Martens et al., 2025). Genotyping is performed once, preferably in a pre-emptive manner, to guide the initial choice of drug and dose (Haidar et al., 2022). TDM should then be routinely considered between weeks four and six of initial treatment, when plasma levels are expected to have stabilised and the antidepressant effect should begin to manifest (Pennazio et al., 2022). In addition, TDM becomes indispensable whenever there is suspicion of therapeutic failure, intolerance or clinically relevant drug–drug interactions (Manubolu et al., 2024).

Complementing this, Yuan et al. (2025) validated and updated paroxetine therapeutic reference ranges (20–65 ng/mL) in Han Chinese patients and showed that CYP2D6 activity score is a major determinant of plasma concentrations. Their prediction models for remission and drug levels illustrate how pharmacogenetic information and TDM can be combined to refine dose individualization, which is consistent with the two-step genotype + TDM framework proposed in our review.

This integrated approach—using genotype to define the initial strategy and TDM to confirm and refine the phenotype—substantially enhances the clinical operability of pharmacogenetic guidelines, making the personalization of antidepressant therapy a tangible practice grounded in objective evidence (Cuvelier et al., 2023; Ter Hark et al., 2025).

**FIGURE 5:**
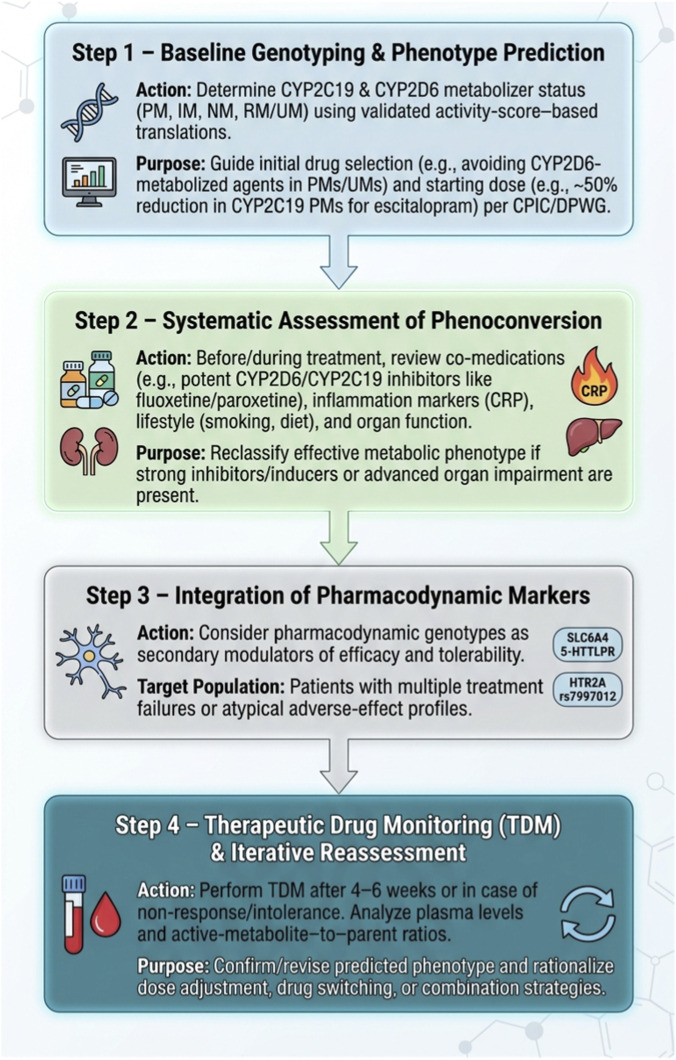
Proposed algorithm integrating genotype, phenoconversion and therapeutic drug monitoring (TDM) in antidepressant management. The framework begins with CYP2C19/CYP2D6 genotyping to predict baseline metabolic phenotype and guide initial drug/dose selection (Step 1), explicitly incorporates phenoconversion due to co-medications, inflammation, lifestyle factors and organ dysfunction (Step 2), integrates pharmacodynamic markers such as SLC6A4 and HTR2A as secondary modulators of efficacy and tolerability (Step 3), and employs TDM for continuous phenotype reassessment and iterative adjustment of dose or drug choice (Step 4). TDM: Therapeutic Drug Monitoring; CYP2C19: Cytochrome P450 2C19; CYP2D6: Cytochrome P450 2D6; PM: Poor Metabolizer; IM: Intermediate Metabolizer; NM: Normal Metabolizer; RM: Rapid Metabolizer; UM: Ultrarapid Metabolizer; CPIC: Clinical Pharmacogenetics Implementation Consortium; DPWG: Dutch Pharmacogenetics Working Group; PPIs: Proton Pump Inhibitors; CRP: C-reactive Protein; SLC6A4: Solute Carrier Family 6 Member 4; and 5-HTTLPR: Serotonin-Transporter-Linked Polymorphic Region.

To synthesize these elements into a practical tool, Figure 5 summarizes a stepwise PK–PD algorithm that starts from genotype-inferred metabolic capacity, explicitly accounts for phenoconversion, incorporates secondary pharmacodynamic markers, and uses TDM for continuous phenotype reassessment during antidepressant management.

## 17 Conclusion

A recent comprehensive review by Bertollo et al. (2025) provides a broad overview of pharmacogenetics in Major Depressive Disorder, integrating insights from genetic predisposition, neurotransmitter pathways, neuroplasticity, and inflammatory responses. Their work, based on a literature search spanning 2000 to 2025, highlights the significant impact of CYP2D6 and CYP2C19 variations on drug metabolism and tolerability, and emphasizes the crucial role of ethnicity and sex-based differences in pharmacogenetic influences on antidepressant response. While Bertollo et al. (2025) offers a holistic perspective, exploring a wider array of genes including BDNF, TrkB, VEGFA, TNF-α, IL-6, and CRHR1, and discussing broader aspects like GWAS findings and detailed genotype frequencies across diverse populations, our manuscript distinguishes itself by offering a more focused and in-depth analysis of the interplay between the specific pharmacokinetic genes (CYP2C19 and CYP2D6) and pharmacodynamic genes (SLC6A4 and HTR2A). This targeted approach allows for a deeper exploration of how these four pivotal genes collectively determine antidepressant efficacy and tolerability, particularly through the lens of recent meta-analyses and the critical concept of phenoconversion, which is central to our discussion. By concentrating on these key serotonergic and metabolic pathways, our review aims to provide a precise and clinically oriented framework for understanding gene–gene interactions relevant to personalized antidepressant therapy, rather than a fully validated prescribing algorithm.

The collective evidence presented throughout this work underscores that genetic variability is a major determinant of antidepressant efficacy, tolerability, and safety, positioning pharmacogenetics as a cornerstone of modern precision psychiatry. The interplay between pharmacokinetic genes—notably *CYP2C19* and *CYP2D6*—and pharmacodynamic genes such as *SLC6A4* (the serotonin transporter) and *HTR2A* (the serotonin 2A receptor) provides a comprehensive framework to explain the wide heterogeneity observed in patient responses to selective serotonin reuptake inhibitors (SSRIs). Empirical evidence from large cohort studies (Li et al., 2024; Kee et al., 2023) and meta-analyses reinforces that genetic differences in drug-metabolizing enzymes directly influence plasma concentrations and clinical outcomes, with poor metabolizers tending toward higher exposure and potential toxicity, and ultrarapid metabolizers often demonstrating underexposure and poor therapeutic response.

Beyond hepatic metabolism, variations in *SLC6A4* and *HTR2A* illustrate how genetic factors modulate central serotonergic signaling and receptor responsiveness, thus shaping antidepressant efficacy and side-effect profiles. Importantly, these findings reveal that effective treatment personalization requires simultaneous integration of both pharmacodynamic and pharmacokinetic markers, a dual perspective that bridges molecular genetics with clinical practice. While current guidelines have successfully implemented genotype-based dose adjustments for *CYP2D6* and *CYP2C19*, there remains a crucial need for meta-analytic consolidation of data on *SLC6A4* and *HTR2A*, particularly stratified by ancestry, sex, and environmental context, to enhance predictive precision and cross-population applicability.

Overall, this synthesis emphasizes that pharmacogenetic testing can transform antidepressant therapy—optimizing drug selection and initial dosing, minimizing adverse reactions, and improving remission rates. The next phase of innovation lies in expanding genetic panels to encompass polymorphisms in novel neurobiological pathways (e.g., glutamatergic and inflammatory mechanisms) and integrating multi-omic data into clinical decision-support systems. By doing so, pharmacogenetics will evolve from an adjunctive diagnostic tool into a fully embedded component of personalized, evidence-driven psychiatry—ultimately offering safer, more effective, and cost-efficient treatments for depressive disorders.

## References

[B1] Adiukwu F. Adesokun O. Essien E. Yalcin N. Ransing R. Nagendrappa S. (2023). Pharmacogenetic testing in psychiatry: perspective on clinical utility. Asian J. Psychiatr. 86, 103674. 10.1016/j.ajp.2023.103674 37327563

[B3] Anderson I. M. (2000). Selective serotonin reuptake inhibitors versus tricyclic antidepressants: a meta-analysis of efficacy and tolerability. J. Affect Disord. 58 (1), 19–36. 10.1016/s0165-0327(99)00092-0 10760555

[B4] Arnone D. Omar O. Arora T. Östlundh L. Ramaraj R. Javaid S. (2023). Effectiveness of pharmacogenomic tests including CYP2D6 and CYP2C19 genomic variants for guiding the treatment of depressive disorders: systematic review and meta-analysis of randomised controlled trials. Neurosci. Biobehav Rev. 144, 104965. 10.1016/j.neubiorev.2022.104965 36463971

[B5] Asp M. Holck A. Green H. Westrin Å. Reis M. (2025). CYP2D6 UM phenotype is not related to suicide attempts in depressive patients in secondary psychiatric care. Nord. J. Psychiatry 79 (7), 556–563. 10.1080/08039488.2025.2553573 40891234

[B6] Baba S. M. Pandith A. A. Shah Z. A. Geelani S. A. Mir M. M. Bhat J. R. (2021). Impact of ABCB1 gene (C3435T/A2677G) polymorphic sequence variations on the outcome of patients with chronic myeloid leukemia and acute lymphoblastic leukemia in Kashmiri population: a case-control study. Indian J. Hematol. Blood Transfus. 37 (1), 21–29. 10.1007/s12288-020-01289-6 33707832 PMC7900282

[B7] Bagshaw A. T. M. (2017). Functional mechanisms of microsatellite DNA in eukaryotic genomes. Genome Biol. Evol. 9 (9), 2428–2443. 10.1093/gbe/evx164 28957459 PMC5622345

[B8] Bahramali E. Firouzabadi N. Yavarian I. Shayesteh M. R. Erfani N. Shoushtari A. A. (2016). Influence of ACE gene on differential response to sertraline versus fluoxetine in patients with major depression: a randomized controlled trial. Eur. J. Clin. Pharmacol. 72 (9), 1059–1064. 10.1007/s00228-016-2079-0 27262302

[B9] Baldwin D. S. Reines E. H. Guiton C. Weiller E. (2007). Escitalopram therapy for major depression and anxiety disorders. Ann. Pharmacother. 41, 1583–1592. 10.1345/aph.1K089 17848424

[B10] Baum M. L. Widge A. S. Carpenter L. L. McDonald W. M. Cohen B. M. Nemeroff C. B. (2024). American psychiatric association (APA) workgroup on biomarkers and novel treatments. Pharmacogenomic clinical support tools for the treatment of depression. Am. J. Psychiatry 181 (7), 591–607. 10.1176/appi.ajp.20230657 38685859

[B11] Bertilsson L. (2007). Metabolism of antidepressant and neuroleptic drugs by cytochrome P450s: clinical and interethnic aspects. Clin. Pharmacol. Ther. 82, 606–609. 10.1038/sj.clpt.6100358 17898711

[B12] Bertollo A. G. Mocelin R. Ignácio Z. M. (2025). Pharmacogenetics and the response to antidepressants in major depressive disorder. Pharm. (Basel) 18 (9), 1360. 10.3390/ph18091360 41011229 PMC12472883

[B13] Berton O. Nestler E. J. (2021). New approaches to antidepressant drug discovery: beyond monoamines. Nat. Rev. Neurosci. 22 (4), 269–282. 10.1038/nrn1846 16429123

[B14] Beunk L. Nijenhuis M. Soree B. de Boer-Veger N. J. Buunk A. M. Guchelaar H. J. (2024). Dutch pharmacogenetics working Group (DPWG) guideline for the gene-drug interaction between CYP2D6, CYP3A4 and CYP1A2 and antipsychotics. Eur. J. Hum. Genet. 32 (3), 278–285. 10.1038/s41431-023-01347-3 37002327 PMC10923774

[B15] Björkman H. (2024). Personalized medicine approaches in clinical pharmacology: integrating pharmacogenomics and therapeutic drug monitoring. Clin. Pharmacol. Biopharm. 13, 5. 10.4172/2167-065X.1000448

[B150] Bousman C. A. Oomen A. Jessel C. D. Tampi R. R. Forester B. P. Eyre H. A. (2021). Perspectives on the clinical use of pharmacogenetic testing in late-life mental healthcare: a survey of the american association of geriatric psychiatry membership. Am. J. Geriatr. Psychiatry 30 (5), 560–571. 10.1016/j.jagp.2021.09.013 34740522

[B16] Bousman C. A. Stevenson J. M. Ramsey L. B. Sangkuhl K. Hicks J. K. Strawn J. R. (2023). Clinical pharmacogenetics implementation consortium (CPIC) guideline for CYP2D6, CYP2C19, CYP2B6, SLC6A4, and HTR2A genotypes and serotonin reuptake inhibitor antidepressants. Clin. Pharmacol. Ther. 114 (1), 51–68. 10.1002/cpt.2903 37032427 PMC10564324

[B17] Brouwer J. M. J. L. Nijenhuis M. Soree B. Guchelaar H. J. Swen J. J. van Schaik R. H. N. (2022). Dutch pharmacogenetics working group (DPWG) guideline for the gene-drug interaction between CYP2C19 and CYP2D6 and SSRIs. Eur. J. Hum. Genet. 30 (10), 1114–1120. 10.1038/s41431-021-01004-7 34782755 PMC9553948

[B18] Brown L. C. Stanton J. D. Bharthi K. Maruf A. A. Müller D. J. Bousman C. A. (2022). Pharmacogenomic testing and depressive symptom remission: a systematic review and meta-analysis of prospective, controlled clinical trials. Clin. Pharmacol. Ther. 112 (6), 1303–1317. 10.1002/cpt.2748 36111494 PMC9827897

[B19] Calleja S. Zubiaur P. Ochoa D. Villapalos-García G. Mejia-Abril G. Soria-Chacartegui P. (2023). Impact of polymorphisms in *CYP* and *UGT* enzymes and *ABC* and *SLCO1B1* transporters on the pharmacokinetics and safety of desvenlafaxine. Front. Pharmacol. 14, 1110460. 10.3389/fphar.2023.1110460 36817149 PMC9934922

[B20] Campos A. I. Byrne E. M. Mitchell B. L. Wray N. R. Lind P. A. Licinio J. (2022). Impact of CYP2C19 metaboliser status on SSRI response: a retrospective study of 9500 participants of the Australian genetics of depression study. Pharmacogenomics J. 22 (2), 130–135. 10.1038/s41397-022-00267-7 35094016 PMC8975743

[B21] Canli T. Lesch K. P. (2007). Long story short: the serotonin transporter in emotion regulation and social cognition. Nat. Neurosci. 10 (9), 1103–1109. 10.1038/nn1964 17726476

[B22] Carvalho Júnior E. Trevisan M. (2021). Psicofarmacologia dos antidepressivos. Braz J. Dev. 7 (11), 107269–107282. 10.34117/bjdv7n11-388

[B23] Caspi A. Sugden K. Moffitt T. E. Taylor A. Craig I. W. Harrington H. (2003). Influence of life stress on depression: moderation by a polymorphism in the 5-HTT gene. Science 301 (5631), 386–389. 10.1126/science.1083968 12869766

[B24] Castro R. S. Souza B. F. Reis M. F. Lalucci MPPS (2024). Uma revisão integrativa do papel do polimorfismo 5-HTTLPR na predisposição à depressão. Arq. Ciênc Saúde UNIPAR 28 (3), 858–875. 10.25110/arqsaude.v28i3.2024-11378

[B25] Caudle K. E. Sangkuhl K. Whirl-Carrillo M. Swen J. J. Haidar C. E. Klein T. E. (2020). Standardizing CYP2D6 genotype to phenotype translation: consensus recommendations from the clinical pharmacogenetics implementation consortium and Dutch pharmacogenetics working group. Clin. Transl. Sci. 13 (1), 116–124. 10.1111/cts.12692 31647186 PMC6951851

[B26] Celestin M. N. Musteata F. M. (2021). Impact of changes in free concentrations and drug-protein binding on drug dosing regimens in special populations and disease states. J. Pharm. Sci. 110 (10), 3331–3344. 10.1016/j.xphs.2021.05.018 34089711 PMC8458247

[B27] Chai A. B. Callaghan R. Gelissen I. C. (2022). Regulation of P-glycoprotein in the brain. Int. J. Mol. Sci. 23 (23), 14667. 10.3390/ijms232314667 36498995 PMC9740459

[B28] Chenchula S. Atal S. Uppugunduri C. R. S. (2024). A review of real-world evidence on preemptive pharmacogenomic testing for preventing adverse drug reactions: a reality for future health care. Pharmacogenomics J. 24 (2), 9. 10.1038/s41397-024-00326-1 38490995 PMC10942860

[B29] Chu A. Wadhwa R. (2023). “Selective serotonin reuptake inhibitors,” in StatPearls (Treasure Island (FL): StatPearls Publishing). Available online at: https://www.ncbi.nlm.nih.gov/books/NBK558978/ (Accessed July 1, 2025). 32119293

[B30] Cicali E. J. Wiisanen K. (2022). The importance of phenoconversion when using the CYP2D6 genotype in clinical practice. Pharmacogenomics 23 (14), 749–752. 10.2217/pgs-2022-0087 36102178 PMC9490503

[B31] Cipriani A. Furukawa T. A. Salanti G. Chaimani A. Atkinson L. Z. Ogawa Y. (2018). Comparative efficacy and acceptability of 21 antidepressant drugs for the acute treatment of adults with major depressive disorder: a systematic review and network meta-analysis. Focus Am Psychiatr. Publ. 16 (4), 420–429. 10.1176/appi.focus.16407 32021580 PMC6996085

[B32] Cui L. Li S. Wang S. Wu X. Liu Y. Yu W. (2024). Major depressive disorder: hypothesis, mechanism, prevention and treatment. Signal Transduct. Target Ther. 9 (1), 30. 10.1038/s41392-024-01738-y 38331979 PMC10853571

[B33] Cuvelier E. Khazri H. Lecluse C. Hennart B. Amad A. Roche J. (2023). Therapeutic drug monitoring and pharmacogenetic testing as guides to psychotropic drug dose adjustment: an observational study. Pharm. (Basel) 17 (1), 21. 10.3390/ph17010021 38256855 PMC10818858

[B34] de Jong L. M. Boussallami S. Sánchez-López E. Giera M. Tushuizen M. E. Hoekstra M. (2023). The impact of *CYP2C19* genotype on phenoconversion by concomitant medication. Front. Pharmacol. 14, 1201906. 10.3389/fphar.2023.1201906 37361233 PMC10285291

[B35] Dean L. Kane M. (2012). “Codeine therapy and CYP2D6 genotype,” in Medical genetics summaries (Bethesda (MD): National Center for Biotechnology Information US). 28520350

[B36] Del Casale A. Gentile G. Lardani S. Modesti M. N. Arena J. F. Zocchi C. (2025). Investigating DRD2 and HTR2A polymorphisms in treatment-resistant schizophrenia: a comparative analysis with other treatment-resistant mental disorders and the healthy state. Eur. Arch. Psychiatry Clin. Neurosci. 276, 1221–1231. 10.1007/s00406-025-01970-9 39934320 PMC13002770

[B37] Dhieb D. Bastaki K. (2025). Pharmaco-multiomics: a new frontier in precision psychiatry. Int. J. Mol. Sci. 26 (3), 1082. 10.3390/ijms26031082 39940850 PMC11816785

[B38] Díaz-Villamarín X. Martínez-Pérez M. Nieto-Sánchez M. T. Fernández-Varón E. Torres-García A. Blancas I. (2025). Clinical pharmacogenetics: results after implementation of preemptive tests in daily routine. J. Pers. Med. 15 (6), 245. 10.3390/jpm15060245 40559107 PMC12194548

[B39] Ding L. Kshirsagar P. Agrawal P. Murry D. J. (2025). Crossing the blood-brain barrier: innovations in Receptor- and transporter-mediated transcytosis strategies. Pharmaceutics 17 (6), 706. 10.3390/pharmaceutics17060706 40574019 PMC12196388

[B40] Drozda K. Pacanowski M. A. Grimstein C. Zineh I. (2018). Pharmacogenetic labeling of FDA-approved drugs: a regulatory retrospective. JACC Basic Transl. Sci. 3 (4), 545–549. 10.1016/j.jacbts.2018.06.001 30175278 PMC6115648

[B41] Edinoff A. N. Akuly H. A. Hanna T. A. Ochoa C. O. Patti S. J. Ghaffar Y. A. (2021). Selective serotonin reuptake inhibitors and adverse effects: a narrative review. Neurol. Int. 13 (3), 387–401. 10.3390/neurolint13030038 34449705 PMC8395812

[B42] Eyal S. Ke B. Muzi M. Link J. M. Mankoff D. A. Collier A. C. (2010). Regional P-glycoprotein activity and inhibition at the human blood-brain barrier as imaged by positron emission tomography. Clin. Pharmacol. Ther. 87 (5), 579–585. 10.1038/clpt.2010.11 20336065 PMC2939015

[B43] Fabbri C. Tansey K. E. Perlis R. H. Hauser J. Henigsberg N. Maier W. (2018). Effect of cytochrome CYP2C19 metabolizing activity on antidepressant response and side effects: meta-Analysis of data from genome-wide association studies. Eur. Neuropsychopharmacol. 28 (8), 945–954. 10.1016/j.euroneuro.2018.05.009 30135031

[B44] Faraj P. Haslemo T. Tran J. P. Stingl J. Molden E. Hole K. (2024). Combined effect of CYP2C19 and CYP2D6 genotypes on escitalopram serum concentration and its metabolic ratio in a European patient population. Br. J. Clin. Pharmacol. 90 (10), 2630–2637. 10.1111/bcp.16156 38925553

[B45] Frye M. A. Nemeroff C. B. (2024). Pharmacogenomic testing for antidepressant treatment selection: lessons learned and roadmap forward. Neuropsychopharmacology 49 (1), 282–284. 10.1038/s41386-023-01667-4 37550439 PMC10700565

[B46] Ganoci L. Trkulja V. Živković M. Božina T. Šagud M. Lovrić M. (2021). ABCB1, ABCG2 and CYP2D6 polymorphism effects on disposition and response to long-acting risperidone. Prog. Neuropsychopharmacol. Biol. Psychiatry 104, 110042. 10.1016/j.pnpbp.2020.110042 32682874

[B47] García-González X. Cubo E. Simón-Vicente L. Mariscal N. Alcaraz R. Aguado L. (2023). Pharmacogenetics in the treatment of Huntington's disease: review and future perspectives. J. Pers. Med. 13 (3), 385. 10.3390/jpm13030385 36983567 PMC10056055

[B48] Geers L. M. Ochi T. Vyalova N. M. Losenkov I. S. Paderina D. Z. Pozhidaev I. V. (2022). Influence of eight ABCB1 polymorphisms on antidepressant response in a prospective cohort of treatment-free Russian patients with moderate or severe depression: an explorative psychopharmacological study with naturalistic design. Hum. Psychopharmacol. 37 (3), e2826. 10.1002/hup.2826 34788473 PMC9285790

[B49] Gettu N. Saadabadi A. (2022). Aripiprazole. StatPearls. Treasure Island, FL: StatPearls Publishing LLC. 31613519

[B50] Ghaffari Darab M. Hedayati A. Khorasani E. Bayati M. Keshavarz K. (2020). Selective serotonin reuptake inhibitors in major depression disorder treatment: an umbrella review on systematic reviews. Int. J. Psychiatry Clin. Pract. 24 (4), 357–370. 10.1080/13651501.2020.1782433 32667275

[B51] Gillman P. K. (2007). Tricyclic antidepressant pharmacology and therapeutic drug interactions updated. Br. J. Pharmacol. 151 (6), 737–748. 10.1038/sj.bjp.0707253 17471183 PMC2014120

[B52] Gloor Y. Lloret-Linares C. Bosilkovska M. Perroud N. Richard-Lepouriel H. Aubry J. M. (2022). Drug metabolic enzyme genotype-phenotype discrepancy: high phenoconversion rate in patients treated with antidepressants. Biomed. Pharmacother. 152, 113202. 10.1016/j.biopha.2022.113202 35653884

[B53] Gras C. Piras M. Ranjbar S. Grosu C. Girardin F. R. Vandenberghe F. (2025). Influence of CYP2D6 genotypes and phenotypes on the plasma levels and clinical response to aripiprazole. Schizophr. Bull. 14, sbaf076. 10.1093/schbul/sbaf076 40662264 PMC12996889

[B54] Haidar C. E. Crews K. R. Hoffman J. M. Relling M. V. Caudle K. E. (2022). Advancing pharmacogenomics from single-gene to preemptive testing. Annu. Rev. Genomics Hum. Genet. 23, 449–473. 10.1146/annurev-genom-111621-102737 35537468 PMC9483991

[B55] Heils A. Teufel A. Petri S. Stöber G. Riederer P. Bengel D. (1996). Allelic variation of human serotonin transporter gene expression. J. Neurochem. 66 (6), 2621–2624. 10.1046/j.1471-4159.1996.66062621.x 8632190

[B56] Hicks J. K. Bishop J. R. Sangkuhl K. Müller D. J. Ji Y. Leckband S. G. (2015). Clinical pharmacogenetics implementation consortium (CPIC) guideline for CYP2D6 and CYP2C19 genotypes and dosing of selective serotonin reuptake inhibitors. Clin. Pharmacol. Ther. 98 (2), 127–134. 10.1002/cpt.147 25974703 PMC4512908

[B57] Hicks J. K. Sangkuhl K. Swen J. J. Ellingrod V. L. Müller D. J. Shimoda K. (2017). Clinical pharmacogenetics implementation consortium guideline (CPIC) for CYP2D6 and CYP2C19 genotypes and dosing of tricyclic antidepressants: 2016 update. Clin. Pharmacol. Ther. 102 (1), 37–44. 10.1002/cpt.597 27997040 PMC5478479

[B58] Hitchman L. M. Faatoese A. Merriman T. R. Miller A. L. Liau Y. Graham O. E. E. (2022). Allelic diversity of the pharmacogene CYP2D6 in New Zealand Māori and Pacific peoples. Front. Genet. 13, 1016416. 10.3389/fgene.2022.1016416 36313436 PMC9606245

[B59] Hoefel L. P. L. (2023). Análise farmacogenética das variantes do gene CYP2C19 no tratamento antidepressivo de pacientes com Transtorno Depressivo Maior [dissertação]. Porto Alegre. Porto Alegre, Brazil: Universidade Federal do Rio Grande do Sul. Available online at: https://lume.ufrgs.br/handle/10183/288031.

[B60] Huang X. Li C. Li C. Li Z. Li X. Liao J. (2021). CYP2C19 genotyping may provide a better treatment strategy when administering escitalopram in Chinese population. Front. Pharmacol. 12, 730461. 10.3389/fphar.2021.730461 34512354 PMC8429954

[B61] Ingelman-Sundberg M. Molden E. (2025). Therapeutic drug monitoring, liquid biopsies or pharmacogenomics for prediction of human drug metabolism and response. Br. J. Clin. Pharmacol. 91 (6), 1569–1579. 10.1111/bcp.16048 38523083 PMC12122132

[B62] Islam F. Marshe V. S. Magarbeh L. Frey B. N. Milev R. V. Soares C. N. (2022). Effects of CYP2C19 and CYP2D6 gene variants on escitalopram and aripiprazole treatment outcome and serum levels: results from the CAN-BIND 1 study. Transl. Psychiatry 12 (1), 366. 10.1038/s41398-022-02124-4 36068210 PMC9448818

[B63] Jallaq S. A. Verba M. Strawn J. R. Martin L. J. DelBello M. P. Ramsey L. B. (2021). CYP2D6 phenotype influences aripiprazole tolerability in pediatric patients with mood disorders. J. Child. Adolesc. Psychopharmacol. 31 (1), 56–62. 10.1089/cap.2020.0058 32845723 PMC8255312

[B64] Jang Y. J. Kim D. K. Lim S. W. Hong E. (2025). Impact of CYP2C19 phenotype on escitalopram response in geriatrics: based on physiologically-based pharmacokinetic modeling and clinical observation. Clin. Pharmacol. Ther. 117 (3), 826–835. 10.1002/cpt.3537 39717930

[B65] Joković D. Milosavljević F. Stojanović Z. Šupić G. Vojvodić D. Uzelac B. (2022). CYP2C19 slow metabolizer phenotype is associated with lower antidepressant efficacy and tolerability. Psychiatry Res. 312, 114535. 10.1016/j.psychres.2022.114535 35398660

[B66] Karg K. Burmeister M. Shedden K. Sen S. (2011). The serotonin transporter promoter variant (5-HTTLPR), stress, and depression meta-analysis revisited: evidence of genetic moderation. Arch. Gen. Psychiatry 68 (5), 444–454. 10.1001/archgenpsychiatry.2010.189 21199959 PMC3740203

[B67] Kato M. Serretti A. (2010). Review and meta-analysis of antidepressant pharmacogenetic findings in major depressive disorder. Mol. Psychiatry 15 (5), 473–500. 10.1038/mp.2008.116 18982004

[B68] Kee P. S. Maggo S. D. S. Kennedy M. A. Chin P. K. L. (2023). The pharmacogenetics of CYP2D6 and CYP2C19 in a case series of antidepressant responses. Front. Pharmacol. 14, 1080117. 10.3389/fphar.2023.1080117 36895946 PMC9988947

[B69] Kehinde O. Ramsey L. B. Gaedigk A. Oni-Orisan A. (2023). Advancing CYP2D6 pharmacogenetics through a pharmacoequity lens. Clin. Pharmacol. Ther. 114 (1), 69–76. 10.1002/cpt.2890 36924260

[B70] Kendrick T. Taylor D. Johnson C. F. (2019). Which first-line antidepressant? Br. J. Gen. Pract. 69 (680), 114–115. 10.3399/bjgp19X701405 30819733 PMC6400617

[B71] Kobayashi K. Chiba K. Yagi T. Shimada N. Taniguchi T. Horie T. (1997). Identification of cytochrome P450 isoforms involved in citalopram N-demethylation by human liver microsomes. J. Pharmacol. Exp. Ther. 280 (2), 927–933. 9023308

[B151] Köhler-Forsberg O. Stiglbauer V. Brasanac J. Chae W. R. Wagener F. Zimbalski K. (2023). Efficacy and safety of antidepressants in patients with comorbid depression and medical diseases: an umbrella systematic review and meta-analysis. JAMA Psychiatry 80 (12), 1196–1207. 10.1001/jamapsychiatry.2023.2983 37672261 PMC10483387

[B72] Leichsenring F. Steinert C. Rost F. Abbass A. Heim N. Ioannidis J. P. A. (2023). A critical assessment of NICE guidelines for treatment of depression. World Psychiatry 22 (1), 43–45. 10.1002/wps.21039 36640399 PMC9840485

[B73] Lense X. M. Hiemke C. Funk C. S. M. Havemann-Reinecke U. Hefner G. Menke A. (2024). Venlafaxine's therapeutic reference range in the treatment of depression revised: a systematic review and meta-analysis. Psychopharmacol. Berl. 241 (2), 275–289. 10.1007/s00213-023-06484-7 37857898 PMC10806172

[B74] Lesch K. P. Bengel D. Heils A. Sabol S. Z. Greenberg B. D. Petri S. (1996). Association of anxiety-related traits with a polymorphism in the serotonin transporter gene regulatory region. Science 274 (5292), 1527–1531. 10.1126/science.274.5292.1527 8929413

[B75] Li D. Pain O. Fabbri C. Wong W. L. E. Lo C. W. H. Ripke S. (2024). Metabolic activity of CYP2C19 and CYP2D6 on antidepressant response from 13 clinical studies using genotype imputation: a meta-analysis. Transl. Psychiatry 14 (1), 296. 10.1038/s41398-024-02981-1 39025838 PMC11258238

[B76] Liang W. S. Beaulieu-Jones B. Smalley S. Snyder M. Goetz L. H. Schork N. J. (2024). Emerging therapeutic drug monitoring technologies: considerations and opportunities in precision medicine. Front. Pharmacol. 15, 1348112. 10.3389/fphar.2024.1348112 38545548 PMC10965556

[B77] Liao Y. Sun Y. Guo J. Kang Z. Sun Y. Zhang Y. (2024). Precision medicine to enhance depression and anxiety outcome consortium. Dose adjustment of paroxetine based on CYP2D6 activity score inferred metabolizer status in Chinese Han patients with depressive or anxiety disorders: a prospective study and cross-ethnic meta-analysis. EBioMedicine 104, 105165. 10.1016/j.ebiom.2024.105165 38776596 PMC11141156

[B79] Maggo S. Kennedy M. A. Barczyk Z. A. Miller A. L. Rucklidge J. J. Mulder R. T. (2019). Common CYP2D6, CYP2C9, and CYP2C19 gene variants, health anxiety, and neuroticism are not associated with self-reported antidepressant side effects. Front. Genet. 10, 1199. 10.3389/fgene.2019.01199 31850065 PMC6901912

[B80] Manubolu K. Gubbala Y. D. Methukumelli T. (2024). “Therapeutic drug monitoring,” in A short guide to clinical pharmacokinetics. Editors Manubolu K. Peeriga R. Chandrasekhar K. B. (Singapore: Springer). 10.1007/978-981-97-4283-7_4

[B81] Marasine N. R. Sankhi S. Lamichhane R. Marasini N. R. Dangi N. B. (2021). Use of antidepressants among patients diagnosed with depression: a scoping review. Biomed. Res. Int. 2021, 6699028. 10.1155/2021/6699028 33791379 PMC7984896

[B82] Martens M. J. Lian Q. Geller N. L. Leifer E. S. Logan B. R. (2025). Sequential monitoring of time-to-event safety endpoints in clinical trials. Clin. Trials 22 (3), 267–278. 10.1177/17407745241304119 40396502 PMC12096354

[B83] McMahon F. J. Buervenich S. Charney D. Lipsky R. Rush A. J. Wilson A. F. (2006). Variation in the gene encoding the serotonin 2A receptor is associated with outcome of antidepressant treatment. Am. J. Hum. Genet. 78 (5), 804–814. 10.1086/503820 16642436 PMC1474035

[B84] Mhandire D. Z. Goey A. K. L. (2022). The value of pharmacogenetics to reduce drug-related toxicity in cancer patients. Mol. Diagn Ther. 26 (2), 137–151. 10.1007/s40291-021-00575-x 35113367 PMC8975257

[B86] Mrazek D. A. Biernacka J. M. O'Kane D. J. Black J. L. Cunningham J. M. Drews M. S. (2011). CYP2C19 variation and citalopram response. Pharmacogenet Genomics 21 (1), 1–9. 10.1097/fpc.0b013e328340bc5a 21192344 PMC3090085

[B87] Nahid N. A. Johnson J. A. (2022). CYP2D6 pharmacogenetics and phenoconversion in personalized medicine. Expert Opin. Drug Metab. Toxicol. 18 (11), 769–785. 10.1080/17425255.2022.2160317 36597259 PMC9891304

[B88] Naoi M. Maruyama W. Shamoto-Nagai M. Riederer P. (2025). Type A monoamine oxidase; its unique role in mood, behavior and neurodegeneration. J. Neural Transm. (Vienna) 132 (3), 387–406. 10.1007/s00702-024-02866-z 39621110

[B89] Ngcobo N. N. (2025). Influence of ageing on the pharmacodynamics and pharmacokinetics of chronically administered medicines in geriatric patients: a review. Clin. Pharmacokinet. 64 (3), 335–367. 10.1007/s40262-024-01466-0 39798015 PMC11954733

[B90] Niitsu T. Fabbri C. Bentini F. Serretti A. (2013). Pharmacogenetics in major depression: a comprehensive meta-analysis. Prog. Neuropsychopharmacol. Biol. Psychiatry 45, 183–194. 10.1016/j.pnpbp.2013.05.011 23733030

[B91] Nofziger C. Turner A. J. Sangkuhl K. Whirl-Carrillo M. Agúndez J. A. G. Black J. L. (2020). PharmVar GeneFocus: CYP2D6. Clin. Pharmacol. Ther. 107 (1), 154–170. 10.1002/cpt.1643 31544239 PMC6925641

[B92] O'Brien F. E. Clarke G. Fitzgerald P. Dinan T. G. Griffin B. T. Cryan J. F. (2012a). Inhibition of P-glycoprotein enhances transport of imipramine across the blood-brain barrier: microdialysis studies in conscious freely moving rats. Br. J. Pharmacol. 166 (4), 1333–1343. 10.1111/j.1476-5381.2012.01858.x 22250926 PMC3417450

[B93] O'Brien F. E. Dinan T. G. Griffin B. T. Cryan J. F. (2012b). Interactions between antidepressants and P-glycoprotein at the blood-brain barrier: clinical significance of *in vitro* and *in vivo* findings. Br. J. Pharmacol. 165 (2), 289–312. 10.1111/j.1476-5381.2011.01557.x 21718296 PMC3268186

[B94] Omran S. Gan S. H. Teoh S. L. (2026). Pharmacogenomics in drug therapy: global regulatory guidelines for managing high-risk drug reactions. Eur. J. Hum. Genet. 34 (1), 27–36. 10.1038/s41431-025-01950-6 40993225 PMC12815907

[B95] Palatini P. De Martin S. (2016). Pharmacokinetic drug interactions in liver disease: an update. World J. Gastroenterol. 22 (3), 1260–1278. 10.3748/wjg.v22.i3.1260 26811663 PMC4716036

[B152] Panneerchelvam S. Norazmi M. N. (2023). DNA profiling in human identification: from past to present. Malays J. Med. Sci. 30 (6), 5–21. 10.21315/mjms2023.30.6.2 38239252 PMC10793127

[B96] Parshenkov M. Zyryanov S. Rodionova G. Dyakonova A. Shegay P. Kaprin A. (2025). Personalizing antidepressant therapy: integrating pharmacogenomics, therapeutic drug monitoring, and digital tools for improved depression outcomes. J. Pers. Med. 15 (12), 616. 10.3390/jpm15120616 41440979 PMC12733439

[B97] Peña-Martín M. C. García-Berrocal B. Sánchez-Martín A. Marcos-Vadillo E. García-Salgado M. J. Sánchez S. (2022). Ten years of experience support pharmacogenetic testing to guide individualized drug therapy. Pharmaceutics 14 (1), 160. 10.3390/pharmaceutics14010160 35057056 PMC8779486

[B98] Pennazio F. Brasso C. Villari V. Rocca P. (2022). Current status of therapeutic drug monitoring in mental health treatment: a review. Pharmaceutics 14 (12), 2674. 10.3390/pharmaceutics14122674 36559168 PMC9783500

[B99] Piacentino D. Bianchi E. De Donatis D. Florio V. Conca A. (2022). Therapeutic drug monitoring of antidepressants: an underused but potentially valuable tool in primary care. Front. Psychiatry 13, 867840. 10.3389/fpsyt.2022.867840 35422716 PMC9002103

[B100] Pirmohamed M. (2024). Pharmacogenetics for the prescriber. Med. (Abingdon) 52 (1), 11–14. 10.1016/j.mpmed.2023.10.002

[B101] Porcelli S. Fabbri C. Serretti A. (2012). Meta-analysis of serotonin transporter gene promoter polymorphism (5-HTTLPR) association with antidepressant efficacy. Eur. Neuropsychopharmacol. 22 (4), 239–258. 10.1016/j.euroneuro.2011.10.003 22137564

[B102] Poweleit E. A. Taylor Z. L. Mizuno T. Vaughn S. E. Desta Z. Strawn J. R. (2023). Escitalopram and sertraline population pharmacokinetic analysis in pediatric patients. Clin. Pharmacokinet. 62 (11), 1621–1637. 10.1007/s40262-023-01294-8 37755681 PMC11003701

[B103] Pratt V. M. Cavallari L. H. Del Tredici A. L. Gaedigk A. Hachad H. Ji Y. (2021). Recommendations for clinical CYP2D6 genotyping allele selection: a joint consensus recommendation of the association for molecular pathology, college of American pathologists, Dutch pharmacogenetics working group of the royal Dutch pharmacists association, and the european society for pharmacogenomics and personalized therapy. J. Mol. Diagn 23 (9), 1047–1064. 10.1016/j.jmoldx.2021.05.013 34118403 PMC8579245

[B104] Preskorn S. H. (1997). Clinically relevant pharmacology of selective serotonin reuptake inhibitors. An overview with emphasis on pharmacokinetics and effects on oxidative drug metabolism. Clin. Pharmacokinet. 32 (Suppl. 1), 1–21. 10.2165/00003088-199700321-00003 9068931

[B105] Principi N. Petropulacos K. Esposito S. (2023). Impact of pharmacogenomics in clinical practice. Pharm. (Basel). 16 (11), 1596. 10.3390/ph16111596 38004461 PMC10675377

[B106] Pritchard D. Patel J. N. Stephens L. E. McLeod H. L. (2022). Comparison of FDA table of pharmacogenetic associations and clinical pharmacogenetics implementation consortium guidelines. Am. J. Health Syst. Pharm. 79 (12), 993–1005. 10.1093/ajhp/zxac064 35230418 PMC9171570

[B107] Prost R. Płaziński W. (2025). Natural polymorphic variants in the CYP450 superfamily: a review of potential structural mechanisms and functional consequences. Int. J. Mol. Sci. 26 (16), 7797. 10.3390/ijms26167797 40869119 PMC12386198

[B108] Radosavljevic M. Svob Strac D. Jancic J. Samardzic J. (2023). The role of pharmacogenetics in personalizing the antidepressant and anxiolytic therapy. Genes (Basel) 14 (5), 1095. 10.3390/genes14051095 37239455 PMC10218654

[B109] Raghavan R. P. Alexander K. T. Sadasivan S. Parmar C. Kathirvel M. (2026). Genetic variants in liver cirrhosis: classifications, mechanisms, and implications for clinical practice. J. Pers. Med. 16 (1), 29. 10.3390/jpm16010029 41590522 PMC12842787

[B110] Rao N. (2007). The clinical pharmacokinetics of escitalopram. Clin. Pharmacokinet. 46 (4), 281–290. 10.2165/00003088-200746040-00002 17375980

[B111] Reis M. Lundmark J. Bengtsson F. (2003). Therapeutic drug monitoring of racemic citalopram: a 5-year experience in Sweden, 1992-1997. Ther. Drug Monit. 25 (2), 183–191. 10.1097/00007691-200304000-00007 12657912

[B112] Richelson E. (2013). Multi-modality: a new approach for the treatment of major depressive disorder. Int. J. Neuropsychopharmacol. 16 (6), 1433–1442. 10.1017/S1461145712001605 23363735 PMC3670520

[B113] Roberts B. Cooper Z. Lu S. Stanley S. Majda B. T. Collins K. R. L. (2023). Utility of pharmacogenetic testing to optimise antidepressant pharmacotherapy in youth: a narrative literature review. Front. Pharmacol. 14, 1267294. 10.3389/fphar.2023.1267294 37795032 PMC10545970

[B114] Sadee W. Wang D. Hartmann K. Toland A. E. (2023). Pharmacogenomics: driving personalized medicine. Pharmacol. Rev. 75 (4), 789–814. 10.1124/pharmrev.122.000810 36927888 PMC10289244

[B115] Sandritter T. Chevalier R. Abt R. Shakhnovich V. (2023). Pharmacogenetic testing for the pediatric gastroenterologist: actionable drug-gene pairs to know. Pharm. (Basel) 16 (6), 889–2023. 10.3390/ph16060889 37375836 PMC10302140

[B116] Sepúlveda-Lizcano L. Arenas-Villamizar V. V. Jaimes-Duarte E. B. García-Pacheco H. Paredes C. S. Bermúdez V. (2023). Metabolic adverse effects of psychotropic drug therapy: a systematic review. Eur. J. Investig. Health Psychol. Educ. 13 (8), 1505–1520. 10.3390/ejihpe13080110 37623307 PMC10453914

[B117] Serretti A. Kato M. De Ronchi D. Kinoshita T. (2007). Meta-analysis of serotonin transporter gene promoter polymorphism (5-HTTLPR) association with selective serotonin reuptake inhibitor efficacy in depressed patients. Mol. Psychiatry 12 (3), 247–257. 10.1038/sj.mp.4001926 17146470

[B118] Serretti A. Barlati S. Buson L. Menesello V. Magistrali A. Silva R. C. (2025). Longitudinal impact of CYP2D6 and CYP2C19 metabolizer status on antidepressant response: the role of pharmacogenetic mismatch. J. Affect Disord. 395 (Pt A), 120724. 10.1016/j.jad.2025.120724 41260361

[B119] Sharp T. Collins H. (2024). Mechanisms of SSRI therapy and discontinuation. Curr. Top. Behav. Neurosci. 66, 21–47. 10.1007/7854_2023_452 37955823

[B120] Sienkiewicz-Oleszkiewicz B. Wiela-Hojeńska A. (2018). CYP2C19 polymorphism in relation to the pharmacotherapy optimization of commonly used drugs. Pharmazie 73 (11), 619–624. 10.1691/ph.2018.8689 30396378

[B121] Simon G. E. Moise N. Mohr D. C. (2024). Management of depression in adults: a review. JAMA 332 (2), 141–152. 10.1001/jama.2024.5756 38856993

[B122] Singh J. Manginas A. Wilkins G. Santosh P. (2025). A comprehensive analysis examining the role of genetic influences on psychotropic medication response in children. Genes (Basel) 16 (9), 1055. 10.3390/genes16091055 41009999 PMC12469437

[B123] Skinner K. T. Palkar A. M. Hong A. L. (2023). Genetics of *ABCB1* in cancer. Cancers (Basel) 15 (17), 4236. 10.3390/cancers15174236 37686513 PMC10487083

[B125] Subash S. Prasad B. (2024). Age-dependent changes in cytochrome P450 abundance and composition in human liver. Drug Metab. Dispos. 52 (12), 1363–1372. 10.1124/dmd.124.001608 39284705 PMC11585312

[B126] Sutanto H. (2025). Tackling polypharmacy in geriatric patients: is increasing physicians’ awareness adequate? Arch. Gerontol. Geriatr. Plus 2 (3), 100185. 10.1016/j.aggp.2025.100185

[B127] Swen J. J. van der Wouden C. H. Manson L. E. Abdullah-Koolmees H. Blagec K. Blagus T. (2023). A 12-gene pharmacogenetic panel to prevent adverse drug reactions: an open-label, multicentre, controlled, cluster-randomised crossover implementation study. Lancet 401 (10374), 347–356. 10.1016/S0140-6736(22)01841-4 36739136

[B128] Tan Y. Y. Jenner P. Chen S. D. (2022). Monoamine Oxidase-B inhibitors for the treatment of Parkinson's Disease: past, present, and future. J. Park. Dis. 12 (2), 477–493. 10.3233/JPD-212976 34957948 PMC8925102

[B129] Taylor C. Crosby I. Yip V. Maguire P. Pirmohamed M. Turner R. M. (2020). A review of the important role of CYP2D6 in pharmacogenomics. Genes (Basel) 11 (11), 1295. 10.3390/genes11111295 33143137 PMC7692531

[B130] Ter Hark S. E. Kievit W. Hannink G. Vos C. F. Spijker J. van der Meij A. (2025). Genotype-specific tricyclic antidepressant dosing in patients with major depressive disorder: a trial-based economic evaluation. Value Health 28 (11), 1714–1721. 10.1016/j.jval.2025.07.018 40769295

[B131] Tiguman G. M. B. Hoefler R. Lima V. G. Silva M. T. Ribeiro-Vaz I. Galvão T. F. (2023). Prevalência do uso de antidepressivos no Brasil: revisão sistemática com meta-análise. J. bras. psiquiatr. 68 (4) 10.1590/0047-2085000000250

[B132] Tortora M. (2023). Personalized pharmacogenomics: optimizing drug selection and dosage based on genetic variants. J. Mol. Genet. Med. 17, 617. 10.37421/1747-0862.2023.17.617

[B133] Uher R. McGuffin P. (2010). The moderation by the serotonin transporter gene of environmental adversity in the etiology of depression: 2009 update. Mol. Psychiatry 15 (1), 18–22. 10.1038/mp.2009.123 20029411

[B134] ul Amin Mohsin N. Farrukh M. Shahzadi S. Irfan M. (2024). Drug metabolism: phase I and phase II metabolic pathways. Pharm. Sci. IntechOpen. 10.5772/intechopen.112854

[B135] van Poelgeest E. P. Pronk A. C. Rhebergen D. van der Velde N. (2021). Depression, antidepressants and fall risk: therapeutic dilemmas-a clinical review. Eur. Geriatr. Med. 12 (3), 585–596. 10.1007/s41999-021-00475-7 33721264 PMC8149338

[B136] van Westrhenen R. Aitchison K. J. Ingelman-Sundberg M. Jukić M. M. (2020). Pharmacogenomics of antidepressant and antipsychotic treatment: how far have we got and where are we going? Front. Psychiatry 11, 94. 10.3389/fpsyt.2020.00094 32226396 PMC7080976

[B138] Veenstra-VanderWeele J. Anderson G. M. Cook E. H. Jr (2000). Pharmacogenetics and the serotonin system: initial studies and future directions. Eur. J. Pharmacol. 410 (2-3), 165–181. 10.1016/s0014-2999(00)00814-1 11134668

[B153] von Moltke L. L. Greenblatt D. J. Giancarlo G. M. Granda B. W. Harmatz J. S. Shader R. I. (2001). Escitalopram (S-citalopram) and its metabolites in vitro: cytochromes mediating biotransformation, inhibitory effects, and comparison to R-citalopram. Drug Metab. Dispos. 29 (8), 1102–1109. 11454728

[B139] Wang Y. Shi J. Dai D. Cai J. Wang S. Hong Y. (2022). Evaluation of commonly used cardiovascular drugs in inhibiting vonoprazan metabolism *in vitro* and *in vivo* . Front. Pharmacol. 13, 909168. 10.3389/fphar.2022.909168 36052128 PMC9424819

[B140] Wong W. L. E. Fabbri C. Laplace B. Li D. van Westrhenen R. Lewis C. M. (2023). The effects of CYP2C19 genotype on proxies of SSRI antidepressant response in the UK biobank. Pharm. (Basel) 16 (9), 1277. 10.3390/ph16091277 37765085 PMC10535191

[B141] Yamada M. Yasuhara H. (2004). Clinical pharmacology of MAO inhibitors: safety and future. Neurotoxicology 25 (1-2), 215–221. 10.1016/S0161-813X(03)00097-4 14697896

[B142] Yan Y. Wang J. Wang D. Luan Y. Sun Q. Chen C. (2025). Impact of CYP2C19 genotype on sertraline and escitalopram exposure and antidepressant switching rate: a retrospective cohort study of Chinese Han patients. Clin. Pharmacol. Ther. 118 (3), 632–641. 10.1002/cpt.3707 40346857

[B143] Yoo H. D. Lee Y. B. (2011). Interplay of pharmacogenetic variations in ABCB1 transporters and cytochrome P450 enzymes. Arch. Pharm. Res. 34 (11), 1817–1828. 10.1007/s12272-011-1104-1 22139683

[B144] Yuan R. Liao Y. Lu X. Kang Z. Guo J. Zhang Y. (2025). Advancing paroxetine treatment in depression: predicting remission and plasma concentration, and validating and updating therapeutic reference ranges. Transl. Psychiatry 15 (1), 321. 10.1038/s41398-025-03503-3 40877220 PMC12394657

[B145] Zelek-Molik A. Litwa E. (2025). Trends in research on novel antidepressant treatments. Front. Pharmacol. 16, 1544795. 10.3389/fphar.2025.1544795 39931695 PMC11807967

[B146] Zhiganova T. A. Radkova E. A. (2025). Genetic variability of CYP2D6, CYP2C19, and CYP1A2 in patients with treatment resistance to antipsychotics and antidepressants. Folia Med. Plovdiv. 67 (4). 10.3897/folmed.67.e149527 40884146

[B147] Zhou Y. Ingelman-Sundberg M. Lauschke V. M. (2017). Worldwide distribution of cytochrome P450 alleles: a meta-analysis of population-scale sequencing projects. Clin. Pharmacol. Ther. 102 (4), 688–700. 10.1002/cpt.690 28378927 PMC5600063

